# Synthesis, Characterization, and Biological Evaluation of Meldrum’s Acid Derivatives: Dual Activity and Molecular Docking Study

**DOI:** 10.3390/ph16020281

**Published:** 2023-02-13

**Authors:** Syed Nasir Abbas Bukhari, Mohamed Abdelwahab Abdelgawad, Naveed Ahmed, Muhammad Wahab Amjad, Muhammad Ajaz Hussain, Mervat A. Elsherif, Hasan Ejaz, Nasser H. Alotaibi, Ignjat Filipović, Nenad Janković

**Affiliations:** 1 Department of Pharmaceutical Chemistry, College of Pharmacy, Jouf University, Sakaka 72388, Al Jouf, Saudi Arabia; 2 Department of Pharmaceutics, College of Pharmacy, Jouf University, Sakaka 72388, Al Jouf, Saudi Arabia; 3 Center for Ultrasound Molecular Imaging and Therapeutics, Pittsburgh Heart, Lung, Blood and Vascular Medicine Institute, University of Pittsburgh, Pittsburgh, PA 15260, USA; 4 Centre for Organic Chemistry, School of Chemistry, University of the Punjab, Lahore 54590, Pakistan; 5 Chemistry Department, College of Science, Jouf University, Sakaka 72388, Al Jouf, Saudi Arabia; 6 Department of Clinical Laboratory Sciences, College of Applied Medical Sciences, Jouf University, Sakaka 72388, Al Jouf, Saudi Arabia; 7 Department of Clinical Pharmacy, College of Pharmacy, Jouf University, Sakaka 72388, Al Jouf, Saudi Arabia; 8 University of Kragujevac, Faculty of Science, Radoja Domanovića 12, 34000 Kragujevac, Serbia; 9 University of Kragujevac, Department of Science, Institute for Information Technologies Kragujevac, Jovana Cvijića bb, 34000 Kragujevac, Serbia

**Keywords:** Meldrum’s acid, vanillin, antimicrobial activity, anticancer activity, gyrase B, topoisomerase II

## Abstract

In the presented study, eight novel Meldrum’s acid derivatives containing various vanillic groups were synthesized. Vanillidene Meldrum’s acid compounds were tested against different cancer cell lines and microbes. Out of nine, three showed very good biological activity against *E. coli*, and HeLa and A549 cell lines. It is shown that the *O*-alkyl substituted derivatives possessed better antimicrobial and anticancer activities in comparison with the *O*-acyl ones. The decyl substituted molecule (**3i**) has the highest activity against *E. coli* (MIC = 12.4 μM) and cancer cell lines (HeLa, A549, and LS174 = 15.7, 21.8, and 30.5 μM, respectively). The selectivity index of **3i** is 4.8 (HeLa). The molecular docking study indicates that compound **3i** showed good binding affinity to DNA, *E. coli* Gyrase B, and topoisomerase II beta. The covalent docking showed that **3i** was a Michael acceptor for the nucleophiles Lys and Ser. The best E_b_ was noted for the topoisomerase II beta-LYS482-**3i** cluster.

## 1. Introduction

Meldrum’s acid presents a compound that has an active methylene group, which was discovered by Meldrum [1]. Therefore, Meldrum proposed the wrong chemical structure. Four decades later, the structure of Meldrum’s acid was solved by Davidson [2]. Its carbonyl function easily can be attacked predominately by oxygen or nitrogen nucleophiles. The C5 position is involved in electrophilic substitution. At high-temperature regimes, Meldrum’s acid and its derivatives could suffer from a fragmentation process that leads to reactive ketene formation [3]. Although it was discovered more than a century ago, its use continues to attract the attention of synthetic chemists. Electrophilicity, dienophilicity, and its relatively low acidity (pKa = 4.9) make Meldrum’s acid attractive and quite useful for a wide number of synthetic manipulations such as heterocycles synthesis, Friedel-Crafts acylation, total synthesis, alkylidene and asymmetric synthesis, domino reactions, and catalytic additions [4,5]. Furthermore, Meldrum’s acid represents an essential molecule in the synthesis of natural products and analogues [6].

Meldrum’s acid derivatives have a wide spectrum of biological activities such as antibacterial, antifungal, anticancer properties that make them attractive from the pharmaceutical point of view [7]. The good antimalarial activity of vanillidene Meldrum’s acid that was comparable with chloroquine was noted against *P. falciparum* [8]. Some vanillidene Meldrum’s acid showed a high antimicrobial effect against *B. subtilis* and *B. cereus* [9]. Bisarylidene Meldrum’s acid possesses significant activity against *S. aureus*, *B. cereus*, *E. coli*, and *P. aeruginosa* species [10]. Promising antibiotic potential together with fluoroquinolones for the selected Meldrum’s acid arylamino methylene group was achieved. In this communication, authors proved this group’s good applicability to fight with reverse resistance in the future [11]. 5-Arilidene derivatives have been published as a promising generation of novel platelet aggregation inhibitors [12]. The importance of aryilidene Meldrum’s acid derivatives were applied as key intermediates in synthesis of various molecules such as epoxide [13], carboxylic acid [14], oxopyridines [15], aldehydes [16], and monoalkyl derivatives [17]. In addition, the presence of an α,β-unsaturated carbonyl function is important in the treatment of various diseases [18,19].

Dual-active drugs are a concept noted as an imperative in future drug design. Namely, a good perspective is found in the development of novel drugs that can have double biological behavior (anticancer–antiviral, –antimicrobial, *etc.*) that have the possibility to cure two different diseases [20,21,22,23,24]. To date, there is no evidence about the potential dual activity of Meldrum’s acid derivatives. Our continual attention on the development of dual-active drugs prompted us to make a novel set of vanillidene Meldrum’s acid derivatives. Going forward, antimicrobial and cytotoxicity evaluation were investigated.

## 2. Results

Our initial idea was to synthesize a set of benzylidene Meldrum’s acid derivatives starting from different vanillic aldehydes that have long-chain fragments. The first reaction was tested with 5′-nitrovanilline (**2a**) and Meldrum’s acid (**1**). Applying similar catalytic conditions such as those described in our published paper [9], we isolated product **3a** (79%). In the presence of the complex salt (PhNH_3_)_2_CuCl_4_ as the catalyst in a concentration of 1 mg/mL (0.85 mol%), the reaction was very fast (5 min). Therefore, PTSA (*p*-toluene sulfonic acid) as catalyst (5 and 10 mol%) produced **3a** in lower yield (62%) during 12 h. The same experimental conditions were tested in reactions **2b** and **2f** with **1**. In both cases PTSA gave lower yield in comparison with (PhNH_3_)_2_CuCl_4_. In some cases, yields were significantly lower. For example, employing PTSA the yield of **3f** was 51%, and 75% when (PhNH_3_)_2_CuCl_4_ was loaded. The results prompted us to apply (PhNH_3_)_2_CuCl_4_ (1 mg/mL) as the catalyst in reaction **1** and various *O*-acyl or -alkyl vanillic aldehydes.

**Scheme 1:**
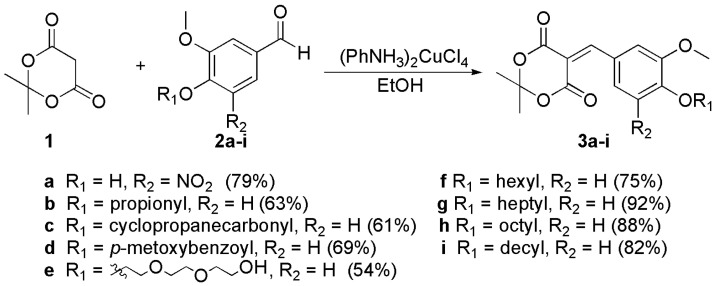
General outline of the synthesis of benzylidene Meldrum’s acid **3a**–**i**.

Applying the catalytic system, good to excellent yields (54–92%) of vanillidene Meldrum’s acid derivatives were obtained (Scheme 1). Almost all products were isolated very easily by filtration. However, there were some problems during the workup of compound **3e**. The increased number of oxygen atoms in the side chain made **3e** very soluble in ethanol. The reaction mixture was evaporated, and upon the addition of the DCM product **3e** was precipitated, filtered, and washed with the DCM. Acyl groups (propionyl, cyclopropanecarbonyl, and *p*-metoxybenzoyl) as structural motifs in **3b**–**d** (61–69%) negatively affected the yield in comparison with long-chain alkyl groups in **3f**–**i** (75–92%).

Proton and carbon NMR spectra of **3a**–**i** samples (cca. 40 mg/mL) were carried out in CDCl_3_ or DMSO-*d_6_* (Figures S1–S18). In every sample, the methyliden proton was the best measure of condensation efficiency. It can be found at approximately 8.35 ppm in proton NMR. In the ^1^H NMR spectrum of **3a** (Figure S1), 1.74 (s), 3.92 (s), 8.14 (s), 8.33 (s), and 8.54 (s) ppm are assigned to 2,2-dimethyl, methoxy, aromatic, and methyliden protons, respectively. The phenolic proton in **3a** is invisible in its NMR, which is presumably caused by the strong electron-withdrawing effect of the nitro group attached to the ortho position. The specific carbon peak located at 155 ppm (Figure S2) was assigned to phenolic carbon, fragment C-OH. Moreover, in all samples, the high-intensity peak at 1.7–1.8 ppm is assigned to the 2,2-dimethyl backbone of Meldrum’s acid fragment. As compared to all carbon NMR, the ^13^C NMR spectrum of **3b**–**d** (Figures S4, S6 and S8) showed new peaks at around 171 ppm that originated from the acyl carbonyl function.

The strong and broad FT-IR peak at around 3204 cm^−1^ is assigned as an overlapped peak of phenolic OH and methyliden function. The band at 2949 cm^−1^ is related to C-H stretching of a chemically non-equivalent double bond functional within **3a**. Two strong bands at 1751 and 1703 cm^−1^ denote the vibration of a carbonyl group from Meldrum’s acid skeleton.

Vanillidene Meldrum’s acid derivatives **3a**–**i** were subjected to biological evaluation. For these experiments, we treated various microbes (*Escherichia coli*, *Bacillus subtilis*, *Staphylococcus aureus*, and *Bacillus cereus*) and cancer cell lines (HeLa, K562, A549, LS174, and PaCa-2). Selectivity was checked towards normal human fibroblast (MRC-5). Streptomycin and cis-platinum (cis-DDP) were chosen as reference standards. The data presented in Table 1 and Table 2 are average antimicrobial and anticancer activities of **3a**–**i** delivered after three independent experiments.

Going deeper inside interactions that should be responsible for antimicrobial or anticancer activity, molecular docking was applied to the most active compounds (**3e**, **3h**, and **3i**). The mentioned compounds were docked to DNA, *Escherichia coli* Gyrase B, and topoisomerase II beta. The energies of binding (E_b_) of **3e**, **3h**, and **3i** are presented in Table 3. The E_b_ of reference compounds ellipticine (for DNA), P3C (for Gyrase B), and etoposide (for topoisomerase II) are −8.98, −7.9, and −14.04 kcal mol^−1^, respectively. Considering these results, the most promising molecule that showed good binding affinity towards reference compounds is **3i**.

## 3. Discussion

The results of antimicrobial testing of **3a**–**i** against selected microbes showed significant activity (Table 1) against Gram-positive (*B. subtilis*, *S. aureus*, and *B. cereus*) and Gram-negative bacteria (*E. coli*). All the tested compounds showed significantly better activity against Gram-negative in comparison with Gram-positive strains. From a structural point of view, vanillidene with the *O*-alkyl fragment (**3e**–**i**) showed to be much more active in contrast to ones with *O*-acyl compounds (**3b**–**d**) against all treated bacteria. The principal reason could be found in the cell wall structural difference. The Gram-positive bacteria cell wall contains peptidoglycans and teichoic acids, while Gram-negative bacteria have lipopolysaccharides and lipopoliproteins as building units of the cell wall [25]. Hence, the presence of a long alkyl chain in structures **3e**–**i** make them more lipophilic, and consequently enables easier passage of these molecules through the cell wall. Out of all nine, the compound with the decyl fragment (**3i**) has the most promising activity against *E. coli* (12.4 μM). Compound **3a** has the lowest activity against all bacterial strains.

**Figure 1:**
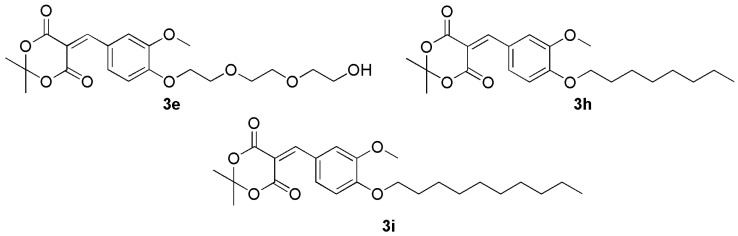
Structures of compounds **3e**, **3h**, and **3i**.

The cytotoxic potential of **3a**–**i** was studied against various cancer and normal cell lines. The results are presented as IC_50_ values in Table 2. Several compounds have good activity, while others possessed a moderate to weak cytotoxic effect. Generally, all nine molecules demonstrated the best activity against A549 cell lines in the range of 21.8–45.7 μM. The best activity was shown by **3i**. In particular, this molecule stands out against HeLa, A549, and LS174 cell lines, with values of 15.7, 21.8, and 30.5, respectively. The achieved IC_50_ values are similar to the ones obtained after treatment of HeLa, A549, and LS174 with cis-DDP. The investigation of MRC-5 cell lines suggests no significant toxicity of **3a**–**g**, while **3h** and **3i** showed moderate toxicity. The three most active compounds possess good selectivity indexes (SI) for HeLa cells (**3h**, **3e**, and **3i**, with values of 2.9, 4.0, and 4.8, respectively). Significant SIs were obtained for A549 cells at 4.3, 2.6, and 3.6 for **3h**, **3e**, and **3i**, respectively. Acyl fragments in structures of **3b**–**d** influenced the obtained IC_50_ value being at least double compared to ones delivered to alkyl substituted vanillidene Meldrum’s acid (**3e–i**). The compounds of interest are **3e**, **3h**, and especially **3i** (Figure 1), which has the highest cytotoxicity and antimicrobial activity against all treated cell lines and bacteria. Considering all results noted during antimicrobial and cytotoxicity testing, we employed molecular docking to investigate the affinity of **3e**, **3h**, and **3i** to DNA, *E. coli* Gyrase B, and topoisomerase II beta at the molecular level.

**Figure 2:**
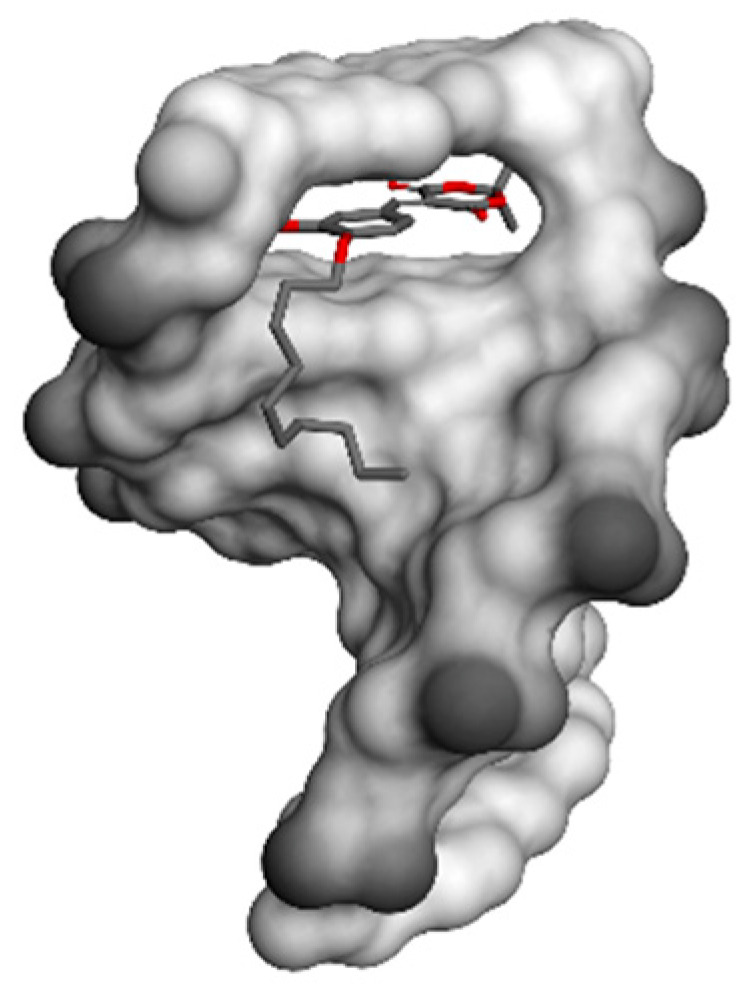
Compound **3i** bound to DNA in intercalative mode.

The selected compounds (**3e**, **3h**, and **3i**,) were computationally evaluated for intercalating ability by docking to a six base pair DNA structure (PDB: 1z3f) against ellipticine, a co-crystalized substrate from the used file [26]. The results are presented in Table 3. Testing of each compound produced results of higher energy when compared against the reference substrate, indicating weaker binding. In each case, the pseudo planar part of the resulting lowest energy structure was packed into an intercalating cavity with slightly different positioning, indicating that each compound may bind in this way. The compound with the longest aliphatic chain (**3i**) showed the most promising results and is displayed in Figure 2.

**Figure 3:**
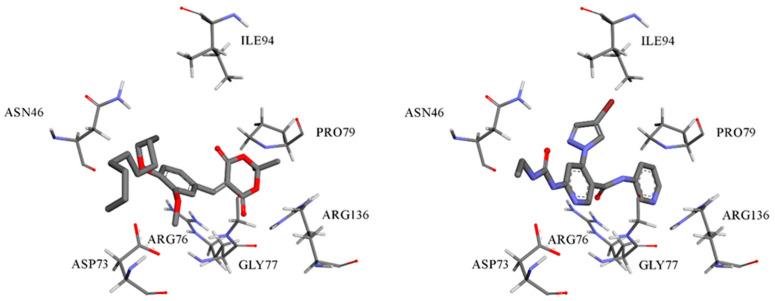
The lowest energy structures that resulted from docking of **3i** to active site of Gyrase B of *E. coli.* (**Left**): **3i**, (**right**): P3C.

It was decided based on our biological tests to computationally examine new compounds for their binding affinity towards enzyme Gyrase B of *E. Coli*. Molecular docking of our compounds was performed on the crystal structure of Gyrase B (PDB:6f86) where the target cavity was the binding place of 4-(4-bromo-1*H*-pyrazol-1-yl)-6-[(ethylcarbamoyl)amino]-*N*-(pyridine-3-yl)pyridine-3-carboxamide (P3C). Since the inhibitory activity of P3C is known (IC_50_ = 0.037 μM [27]), it serves as a good reference for comparison of obtained docking results for new inhibitors. Binding energies are presented in Table 3. Based on the obtained binding energies, we can conclude that most of our compounds would generally show weaker inhibitory activity when compared to P3C, but the result obtained from docking **3i** is comparable to the result displayed by the reference compound. The position of the docked structure for **3i** is similar to the position of PC3, and this similarity is evident from Figure 3 and Figure S19. The difference is, for example, that **3i** does not possess a side group bound to a central aromatic ring like the bromopyrazolyl moiety of P3C, which allows interactions with and in the vicinity of ILE94, while **3i** has a long aliphatic chain that extends much deeper into the cavity of the enzyme than the ethylcarbamoyl group of P3C.

To further examine the potential anticancer application of examined compounds, molecular docking experiments were performed on the crystal structure of type II topoisomerase beta (TOP2β) in a complex with DNA and the anticancer drug etoposide (PDB: 3qx3) [28]. Benzylidene Meldrum’s acid was noted as the key precursor to novel topoisomerase II inhibitors [29]. One of them is α-lapachone, which is approved as a topoisomerase II inhibitor [30,31]. Redocking of etoposide was used to obtain the reference value of binding energy. The main reason for choosing the selected binding site was comparison with the selected substrate (etoposide) that binds in that exact site. Topoisomerase II is symmetric, and there are two identical sides. Docking experiments were conducted on both sides and identical results (geometry and energy of binding) were obtained. For better clarity we present only one of the identical binding modes.

**Figure 4:**
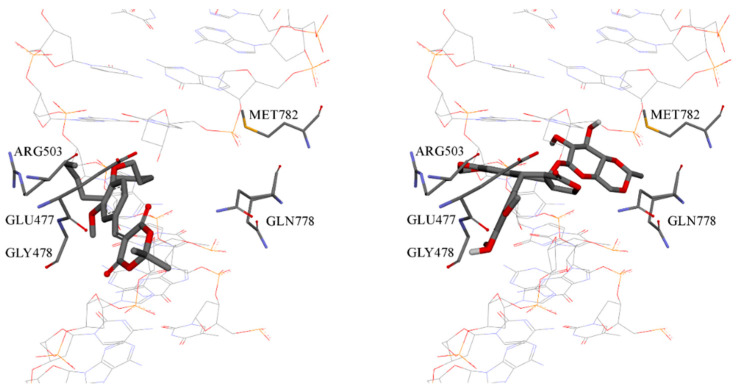
Comparison of binding modes for lowest energy **3i** structure (**left**) and lowest energy etoposide structure (**right**).

The results derived from docking of the examined compounds are compared against the reference value in Table 3. Significantly higher binding energies of tested compounds when compared to etoposide suggest much weaker binding in the same pocket. Upon investigation of structures obtained from the best docking results, it was found that contrary to etoposide, which intercalates with DNA and interacts with the amino acid residues of the enzyme that can be found on both sides of the DNA chain, our compounds position themselves in such a way that only allows interactions with residues from a single side of the chain (Figure 4 and Figure S20).

Docking of covalently bound ligands is important for gaining insight into enzymatic processes and designing superior covalent ligands. AutoDock 4 was used to implement two methods for docking covalently bound ligands: a grid-based method and a modification of the flexible side chain approach [32]. Covalent docking has also been applied to oligopeptidase [33] and proteasome [34] inhibitors and glycoside hydrolyses ligands [35]. Benzylidene Meldrum’s acid was used as a valuable Michael acceptor [36,37]. Consequently, in this paper, covalent docking was employed to simulate the formation of the covalent adduct using the flexible side chain technique proposed by Bianco et al. [32]. This protocol needs to adapt the residue taking part in the covalent bond by attaching the ligand to its side chain; this modified residue is then considered flexible during the docking calculation. Docking was attained for the ligand, keeping the remodeled cysteine and serine residues flexible. This permitted us to sample the torsional flexibility of the ligand within the Gyrase B and topoisomerase II beta. It was shown that the best conformational compatibility between the subjected ligand Michael acceptor (**3i**) and substrate Gyrase B or topoisomerase II beta had residues LYS139 (Gyrase B), LYS482, and SER480 (topoisomerase II beta). The molecular interactions between **3i** and the amino acids lysine and serine in Gyrase B and topoisomerase II beta were estimated by using covalent docking calculations. The obtained results for E_b_ are presented in Table 4. The more negative E_b_ values indicate that the investigated ligand **3i** inhibits the receptor better.

**Figure 5:**
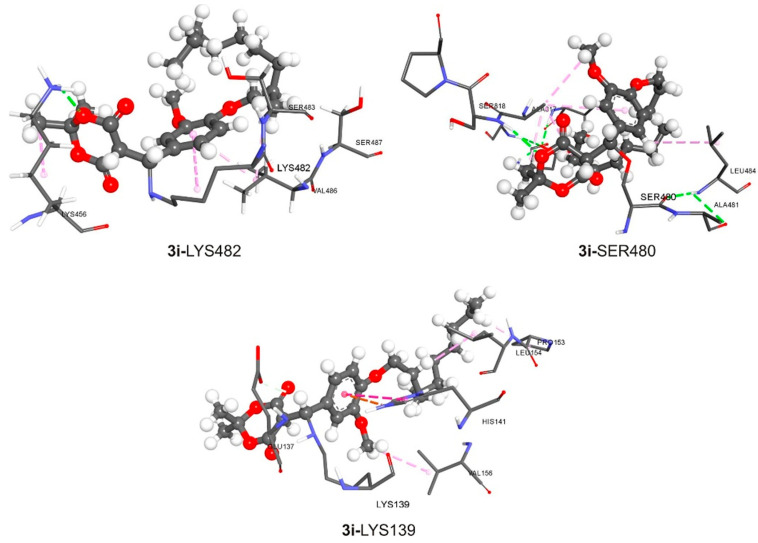
The docking interactions of the most stable conformation of **3i** and investigated receptors.

As shown in Table 4, the investigated ligand strongly binds to the target receptors. The docking analyses revealed that covalent interactions existed between the investigated molecule and target receptors. These interactions occur between **3i** and the amino acids LYS and SER in positions 482 and 480 in the primary structure of the topoisomerase II beta-receptor. On the other hand, **3i** forms the covalent bond with the amino acid LYS139 from Gyrase B (Figure 5). Additionally, the docking results show that several non-covalent interactions occurred between the investigated molecule and target receptors. The important interactions are hydrogen bonds, carbon–hydrogen bonds, alkyl–alkyl, and alkyl–π interactions (Figure 5). The obtained results indicate that the **3i** ligand could act as a potential covalent inhibitor.

The α- and β-lapachones were described as covalent inhibitors of topoisomerase II [38]. The mentioned compounds have also been investigated as irreversible catalytic inhibitors of topoisomerase II [30]. The high affinity of **3i** to form a covalent bond with topoisomerase II beta makes it similar to the aforementioned Meldrum’s acid analogs.

## 4. Materials and Methods

The IR spectra were recorded by a Perkin–Elmer Spectrum One FT-IR spectrometer on a KBr pellet. The NMR spectra of compounds **3a**–**i** were performed in DMSO-*d_6_* and CDCl_3_ with TMS as the internal standard on a Varian Gemini 200 MHz NMR spectrometer (^1^H at 200 and ^13^C at 50 MHz). Abbreviations for the NMR signals that were used are as follows: s = singlet, d = doublet, dd = double of double, t = triplet, and m = multiplet. The ^1^H and ^13^C spectra are given in Supplementary Information (Figures S1–S18).

### 4.1. Synthesis of Benzylidene Meldrum’s Acid Derivatives (***3***)

In the 10 mL round-bottomed flask, Meldrum’s acid (1.5 mmol) was dissolved in 5 mL of absolute ethanol. Selected aldehyde was added at room temperature. Immediately after, (PhNH_3_)_2_CuCl_4_ (1 mg/mL, 0.85 mol%) was loaded as catalyst. Precipitation of products occurred in the interval of 5 to 10 min. One hour later, amorphous powder was filtrated and washed out with 96% ethanol and water. Knoevenagel adducts were isolated in good purity grade for NMR measurements. NMR data for **3a**–**i** are given below.

*5-(4′-hydroxy-3′-methoxy-5′-nitrobenzylidene)-2,2-dimethyl-1,3-dioxane-4,6-dione* **3a**

Green solid; yield: 79% (383 mg); IR ν 3204, 2949, 1751, 1703, 1577, 1541 cm^−1^; ^1^H NMR (200 MHz, DMSO-d_6_) δ 8.54 (s, 1H), 8.33 (s, 1H), 8.14 (s, 1H), 3.92 (s, 3H), 1.74 (s, 6H) ppm; ^13^C NMR (50 MHz, DMSO-d_6_) δ 162.8, 160.0, 155.1, 147.3, 124.2, 121.8, 120.2, 113.5, 104.5, 56.9, 27.2 ppm; ESI-MS (*m*/*z*): [M + Na]^+^ = 346.

*2′-methoxy-4′-((2,2-dimethyl-4,6-dioxo-1,3-dioxan-5-ylidene)methyl)phenyl propionate* **3b**

Light green solid; yield: 63% (316 mg); IR ν 3465, 2991, 2944, 1762, 1733, 1598, 1582, 1512 cm^−1^; ^1^H NMR (200 MHz, CDCl_3_) δ 8.36 (s, 1H), 8.22 (d, *J* = 2.0 Hz, 1H), 7.63–7.51 (m, 1H), 7.15 (d, *J* = 8.2 Hz, 1H), 3.90 (s, 3H), 2.65 (q, *J* = 7.5 Hz, 2H), 1.80 (s, 6H), 1.29 (t, *J* = 7.5 Hz, 3H) ppm; ^13^C NMR (50 MHz, CDCl_3_) δ 171.7, 163.3, 159.8, 157.1, 151.1, 144.6, 130.2, 129.1, 123.0, 116.9, 114.1, 104.5, 56.1, 27.6, 27.4, 9.1 ppm; ESI-MS (*m*/*z*): [M + H]^+^ = 335.

*2′-methoxy-4′-((2,2-dimethyl-4,6-dioxo-1,3-dioxan-5-ylidene)methyl)phenyl cyclopropanecarboxylate* **3c**

Light green solid; yield: 61% (317 mg); IR ν 3469, 2985, 1753, 1733, 1513, 1379 cm^−1^; ^1^H NMR (200 MHz, CDCl_3_) δ 8.36 (s, 1H), 8.22 (d, *J* = 2.0 Hz, 1H), 7.57 (dd, *J* = 8.4, 2.0 Hz, 1H), 7.17 (d, *J* = 8.3 Hz, 1H), 3.91 (s, 3H), 1.96–1.80 (m, 7H), 1.23–1.01 (m, 4H) ppm; ^13^C NMR (50 MHz, CDCl_3_) δ 172.1, 163.3, 159.8, 157.1, 151.2, 144.6, 130.2, 129.1, 123.1, 116.9, 114.1, 104.46, 56.1, 27.6, 12.9, 9.5 ppm; ESI-MS (*m*/*z*): [M + H]^+^ = 347.

*2′-methoxy-4′-((2,2-dimethyl-4,6-dioxo-1,3-dioxan-5-ylidene)methyl)phenyl 4-methoxybenzoate*  **3d**

Green solid; yield: 69% (426 mg); IR ν 3446, 2993, 1732, 1606, 1577 cm^−1^; ^1^H NMR (200 MHz, CDCl_3_) δ 8.40 (s, 1H), 8.26 (d, *J* = 1.8 Hz, 1H), 8.15 (t, *J* = 5.7 Hz, 2H), 7.62 (dd, *J* = 8.3, 1.9 Hz, 1H), 7.30–7.26 (m, 1H), 6.98 (t, *J* = 5.7 Hz, 2H), 3.89 (d, *J* = 1.9 Hz, 6H), 1.81 (s, 6H) ppm; ^13^C NMR (50 MHz, CDCl_3_) δ 164.0, 163.7, 163.3, 159.8, 157.2, 151.4, 144.9, 132.5, 130.2, 129.2, 123.2, 121.1, 117.0, 114.1, 113.9, 104.5, 56.1, 55.5, 27.6 ppm; ESI-MS (*m*/*z*): [M + H]^+^ = 413.

*5-(4′-(2″-(2′″-(2″″-hydroxyethoxy)ethoxy)ethoxy)-3′-methoxybenzylidene)-2,2-dimethyl-1,3-dioxane-4,6-dione* **3e**

Green solid; yield: 54% (332 mg); IR ν 3583, 3439, 2939, 2913, 1745, 1708, 1578, 1559 cm^−1^; ^1^H NMR (200 MHz, CDCl_3_) δ 8.35 (s, 1H), 8.28 (d, *J* = 2.0 Hz, 1H), 7.63 (dd, *J* = 8.6, 2.0 Hz, 1H), 6.97 (d, *J* = 8.5 Hz, 1H), 4.33–4.25 (m, 2H), 3.95–3.85 (m, 5H), 3.76–3.59 (m, 8H), 2.58 (s, 1H), 1.80 (s, 6H) ppm; ^13^C NMR (50 MHz, CDCl_3_) δ 163.9, 160.4, 158.0, 153.9, 148.9, 132.2, 125.2, 116.2, 111.9, 110.7, 104.1, 72.5, 70.9, 70.3, 69.2, 68.5, 61.7, 55.9, 27.5 ppm; ESI-MS (*m*/*z*): [M + H]^+^ = 411.

*5-(4′-hexyloxy-3′-methoxybenzylidene)-2,2-dimethyl-1,3-dioxane-4,6-dione* **3f**

Yellow solid; yield: 75% (407 mg); IR ν 3444, 2956, 2937, 1748, 1713, 1558, 1523 cm^−1^; ^1^H NMR (200 MHz, CDCl_3_) δ 8.36 (s, 1H), 8.29 (d, *J* = 2.1 Hz, 1H), 7.64 (dd, *J* = 8.6, 2.1 Hz, 1H), 6.94 (d, *J* = 8.6 Hz, 1H), 4.13 (t, *J* = 6.9 Hz, 2H), 3.94 (s, 3H), 1.93–1.82 (m, 8H), 1.56–1.26 (m, 6H), 0.94–0.87 (m, 3H) ppm; ^13^C NMR (50 MHz, CDCl_3_) δ 164.1, 160.5, 158.2, 154.5, 148.9, 132.6, 124.7, 116.0, 111.4, 110.2, 104.0, 69.2, 56.0, 31.5, 28.8, 27.5, 25.5, 22.5, 14.0 ppm; ESI-MS (*m*/*z*): [M + H]^+^ = 363.

*5-(4′-heptyloxy-3′-methoxybenzylidene)-2,2-dimethyl-1,3-dioxane-4,6-dione* **3g**

Yellow solid; yield: 92% (519 mg); IR ν 3436, 2949, 2923, 1746, 1708, 1548, 1522 cm^−1^; ^1^H NMR (200 MHz, CDCl_3_) δ 8.36 (s, 1H), 8.29 (d, *J* = 2.1 Hz, 1H), 7.64 (dd, *J* = 8.6, 2.1 Hz, 1H), 6.94 (d, *J* = 8.5 Hz, 1H), 4.13 (t, J = 6.9 Hz, 2H), 3.94 (s, 3H), 2.06–1.77 (m, 8H), 1.77–1.03 (m, 8H), 1.03–0.79 (m, 3H) ppm; ^13^C NMR (50 MHz, CDCl_3_) δ 164.1, 160.5, 158.2, 154.5, 148.9, 132.6, 124.7, 116.1, 111.4, 110.3, 104.0, 69.2, 56.0, 31.7, 29.0, 28.9, 27.5, 25.8, 22.6, 14.1 ppm; ESI-MS (*m*/*z*): [M + H]^+^ = 377.

*5-(3′-methoxy-4′-octyloxybenzylidene)-2,2-dimethyl-1,3-dioxane-4,6-dione* **3h**

Yellow solid; yield: 88% (515 mg); IR ν 3439, 2950, 2930, 1745, 1708, 1549, 1523 cm^−1^; ^1^H NMR (200 MHz, CDCl_3_) δ 8.36 (s, 1H), 8.30 (d, *J* = 2.1 Hz, 1H), 7.63 (d, *J* = 8.5 Hz, 1H), 6.94 (d, *J* = 8.6 Hz, 1H), 4.13 (t, *J* = 6.8 Hz, 2H), 3.94 (s, 3H), 2.05–1.62 (m, 8H), 1.60–1.16 (m, 10H), 0.89 (m, 3H) ppm; ^13^C NMR (50 MHz, CDCl_3_) δ 164.0, 160.5, 158.1, 154.5, 148.9, 132.5, 124.7, 116.1, 111.4, 110.3, 103.9, 69.2, 56.0, 31.7, 29.2, 29.1, 28.8, 27.5, 25.8, 22.6, 14.0 ppm; ESI-MS (*m*/*z*): [M + H]^+^ = 391.

*5-(4′-decyloxy-3′-methoxybenzylidene)-2,2-dimethyl-1,3-dioxane-4,6-dione* **3i**

Yellow solid; yield: 82% (514 mg); IR ν 3439, 2952, 2928, 1746, 1709, 1545, 1521 cm^−1^; ^1^H NMR (200 MHz, CDCl_3_) δ 8.36 (s, 1H), 8.30 (d, *J* = 2.1 Hz, 1H), 7.64 (dd, *J* = 8.6, 2.1 Hz, 1H), 6.94 (d, *J* = 8.6 Hz, 1H), 4.13 (t, *J* = 6.8 Hz, 2H), 3.94 (s, 3H), 2.00–1.68 (m, 8H), 1.62–1.16 (m, 14H), 0.88 (t, *J* = 6.4 Hz, 3H) ppm; ^13^C NMR (50 MHz, CDCl_3_) δ 164.0, 160.5, 158.1, 154.5, 148.9, 132.5, 124.7, 116.0, 111.4, 110.3, 104.0, 69.2, 56.0, 31.9, 29.5, 29.3, 29.3, 28.8, 27.5, 25.8, 22.6, 14.1 ppm; ESI-MS (*m*/*z*): [M + H]^+^ = 419.

### 4.2. Determination of Antimicrobial Activity

The following strains of bacteria were used as test organisms in this study: *Staphylococcus aureus* (ATCC 25923), *Bacillus subtilis* (ATCC 6633), *Bacillus cereus* (ATCC 10987), and *Escherichia coli* (ATCC 25922). All the bacteria used were obtained from the American Type Culture Collection (ATCC). Cultures were stored at 4 °C and subcultured every 15 days. The minimal inhibitory concentration (MIC) was determined by the broth microdilution method using 96-well micro-titer plates [39]. A series of dilutions of the tested compounds was used in the experiment against every microorganism. The starting solutions of tested compounds were obtained by measuring off a certain quantity of the compounds and dissolving it in 5% DMSO. The MIC was determined with resazurin. The inoculated plates were incubated at 37 °C for 24 h. Resazurin is an oxidation–reduction indicator used for the evaluation of microbial growth. It is a blue non-fluorescent dye that becomes pink and fluorescent when reduced to resorufin by oxidoreductases within viable cells. The boundary dilution without any changing in color of resazurin was defined as the MIC for the tested microorganism at a given concentration. As a positive control of growth inhibition, streptomycin was used. A 5% DMSO solution was used as a negative control for the influence of the solvents.

### 4.3. Evaluation of Cytotoxicity

Cell line cells were obtained from the American Type Culture Collection. Human cervical adenocarcinoma HeLa, human chronic myelogenous leukemia K562, non-small cell lung carcinoma A549, human colon carcinoma LS174, pancreatic carcinoma PaCa-2, and normal human lung fibroblast MRC-5 cell lines were grown in RPMI-1640 medium with 10% fetal bovine serum, L-glutamine, and penicillin–streptomycin solution. Cells were plated into the 96-well cell culture plates. Cells were incubated at 37 °C in CO_2_ incubator. Adherent cell lines were incubated for 20 h before addition of tested compounds. Two hours before addition of compounds, K562 cells were seeded into 96-well plates. The tested compounds were applied at five different concentrations ranging from 10 to 300 µM for 72 h. Thereafter, the survival of cells was determined by MTT assay in accordance with the protocol established by Mosmann [40] and Ohno and Abe [41]. The solution of MTT was added to blank and cell samples. The plates were incubated for 5 h at 37 °C, and then 10% solution of sodium dodecyl sulfate was added to the wells. The absorbance was measured at 570 nm using Thermo Scientific Multiskan EX plate reader. Three independent experiments were performed.

### 4.4. Molecular Docking

Molecular docking experiments were prepared using AutoDockTools and were performed using AutoDock 4 [42]. Structures of target molecules were obtained from rscb.org, and were prepared by first removing co-crystalized substrates, ions, and water molecules, followed by calculating Gasteiger charges and removing non-polar hydrogens. Structures of investigated molecules were optimized using PM7 method of MOPAC2016 [43], but Gasteiger charges were calculated for use in docking experiments. Each co-crystalized substrate was re-docked to its original parent molecule to obtain reference value of binding energy and to validate the applied method. Grid maps were calculated using cube-shaped grid boxes 60 pts wide for protein targets and 40 pts wide for DNA target (1 pt = 0.375 A). Grid boxes were centered using coordinates of co-crystalized substrates. Each experiment consisted of 10 hybrid genetic algorithm–local search runs, with 2.5 × 10^7^ energy evaluations per run.

The two-point attractor and flexible side chain approaches [32] are two types of covalent docking methods that can be used with AutoDock 4. Both methods use precalculated grid maps and atom probes to speed up the scoring process. However, they simulate ligand conformations in different ways during the scoring process. In this paper, we used the flexible side chain method. This approach uses tethered docking to mimic the way covalent ligands are bound in the pocket. For this plan to work, the electrophilic center of the ligand must bond to the two nucleophilic atoms at the ends of the protein. By figuring out the right SMARTS pattern, these two atoms are put on top of the right residue atoms in the protein to make the structure that is wanted. The bound ligand is then treated as a flexible residue, and the standard AutoDock 4 method for flexible residues is used to look at its different shapes in the pocket. With the aid of the scripts provided by the AutoDock 4 website [44], the ei ligand and cysteine and serine residues were overlapped. Subsequently, the receptor grid maps were calculated with the AutoGrid4 software, mapping the receptor interaction energies using the ligand atom types as probes. The grid of 60 Å × 60 Å × 60 Å with 0.375 Å spacing was centered on the coordinates of the ligand originally present in the Gyrase B and topoisomerase II beta. Finally, the actual docking was attained for the ei ligand, keeping the cysteine and serine residues as flexible. This permitted the sampling of the torsional flexibility within the receptors.

## 5. Conclusions

In this paper, eight novel Meldrum’s acid derivatives containing vanillic fragments were synthesized under soft reaction conditions in good to excellent yields (54–92%). All compounds showed good to moderate anticancer and antimicrobial activities. Vanillidene Meldrum’s acid derivatives with an *O*-acyl group attached on the vanillic motif have lower activity against cancer cell lines and bacteria. However, the presence of the *O*-alkyl fragment significantly influenced the biological activity, both anticancer and antimicrobial. The most active compound that showed dual activity was **3i**. This molecule contains a decyl chain that is probably responsible for the good activity against *E. coli* (12.4 μM) and cancer cell lines (IC_50_ for HeLa, LS174, and A549: 15.7, 20.8, and 21.8 μM, respectively). Significant dual activity was displayed by compounds **3e** and **3h**. Very good selectivity indices were accomplished with **3e**, **3h** and **3i**. The highest selectivity index was for **3i** against HeLa (4.8), while the next highest was **3h** against A549 cell lines (4.3). In further investigation, the most active compounds (**3e**, **3h**, and **3i**) were subjected to molecular docking. The binding affinities of mentioned compounds to DNA, *E. coli* Gyrase B, and topoisomerase II beta were tested. Compounds **3e**, **3h**, and **3i** showed good non-covalent interactions and consequently good energy of binding. Therefore, **3i** possessed the highest affinity and was subjected to a covalent docking study. Under this investigation, **3i** was played as the Michael acceptor in the reaction with the amino or hydroxy group from Lys and Ser residues, respectively. The lowest binding energy was realized for the topoisomerase II beta-LYS482-**3i** cluster (E_b_ = −7.40 kcal mol^−1^). From molecular docking and experimental outputs, it was shown that **3i** is a molecule of interest. Going forward, we believe that the presented concept and dual-active compounds described herein have bright futures.

## Figures and Tables

**Table 1:** Antimicrobial activity of **3a–i**. MIC values are presented in μM.

	*E. coli*	*B. subtilis*	*S. aureus*	*B. cereus*
**3a**	115.3	-	-	-
**3b**	89.5	175.3	210.5	174.2
**3c**	85.1	180.5	205.8	171.5
**3d**	74.8	169.1	199.6	168.4
**3e**	38.1	133.1	145.1	80.5
**3f**	37.5	48.3	73.9	42.3
**3g**	36.1	45.2	60.4	37.5
**3h**	14.7	31.5	54.2	64.8
**3i**	12.4	29.5	50.9	41.5
streptomycin	0.010	0.003	0.005	0.003

**Table 2:** Cytotoxicity of **3a–i** (MTT in μM).

	HeLa	K562	A549	LS174	PaCa-2	MRC-5
**3a**	126.1 ± 1.45	151.2 ± 3.47	>200	>200	>200	>200
**3b**	101.7 ± 2.14	74.5 ± 1.73	41.2 ± 1.74	104.7 ± 4.62	152.7 ± 4.29	>200
**3c**	84.6 ± 1.84	70.2 ± 1.68	45.7 ± 1.65	99.6 ± 1.44	172.5 ± 5.25	>200
**3d**	94.5 ± 3.15	105.1 ± 0.92	36.2 ± 0.39	118.9 ± 0.95	145.1 ± 2.92	>200
**3e**	37.3 ± 1.20	80.5 ± 2.36	25.9 ± 0.46	38.1 ± 0.41	181.5 ± 3.94	110.9 ± 2.19
**3f**	63.5 ± 0.45	71.9 ± 1.65	39.6 ± 0.74	45.3 ± 0.58	110.5 ± 1.75	144.2 ± 2.84
**3g**	62.6 ± 0. 71	69.5 ± 1.37	35.1 ± 0.27	47.1 ± 1.69	94.2 ± 1.09	139.1 ± 3.07
**3h**	18.2 ± 0.11	73.5 ± 1.43	27.4 ± 0.78	36.5 ± 0.24	90.5 ± 0.83	72.8 ± 0.77
**3i**	15.7 ± 0.28	63.7 ± 2.49	21.8 ± 0.91	30.5 ± 0.35	58.2 ± 0.74	74.6 ± 1.46
cis-DDP	2.36 ± 0.28	5.56±0.23	17.93 ± 0.44	20.8 ± 0.44	25.8 ± 0.65	4.26 ± 0.46

**Table 3:** Energies of binding (E_b_) derived from docking of **3e**, **3h**, and **3i** to DNA (I), *Escherichia coli* Gyrase B (II), and topoisomerase II beta (III).

	E_b_ (kcal mol^−1^)		
	I	II	III
**3e**	−5.61	−5.45	−8.46
**3h**	−6.94	−7.20	−9.37
**3i**	−7.74	−7.52	−9.51

**Table 4:** Binding energies for best docking conformation of **3i** with Gyrase B and topoisomerase II beta.

	E_b_ (kcal mol^−1^)		
	Gyrase B-LYS139	Topoisomerase-LYS482	Topoisomerase-SER480
**3i**	−5.64	−7.40	−5.24

## Data Availability

Data is contained within the article and supplementary material.

## Supplementary Materials

The following supporting information can be downloaded at: https://www.mdpi.com/article/10.3390/ph16020281/s1, Figures S1–S18: NMR spectra of **3a**–**i**.
