# Supplementary material for: Synthesis, Characterization, and Biological Evaluation of Meldrum’s Acid Derivatives: Dual Activity and Molecular Docking Study
Source: Pharmaceuticals (Basel). 2023 Feb 13;16(2):281. doi: 10.3390/ph16020281 (PMC9968196; doi:10.3390/ph16020281)
Supplement: Supplementary file 1 [file pharmaceuticals-16-00281-s001.zip › pharmaceuticals-2154009-supplementary.pdf]

PHARMACEUTICALS

Supplementary Information associated with the paper

Synthesis, characterization, and biological evaluation of Meldrum's acid derivatives: Dual activity and molecular docking study

Syed Nasir Abbas Bukhari,[1*] Mohamed Abdelwahab Abdelgawad,[1] Naveed Ahmed,[2] Muhammad Wahab Amjad,[3] Muhammad Ajaz Hussain,[4] Mervat A. Elsherif,[5] Hasan Ejaz,[6] Nasser H. Alotaibi,[7] Ignjat Filipović,[8] and Nenad Janković[9*]

[1]Department of Pharmaceutical Chemistry, College of Pharmacy, Jouf University, Sakaka, Al Jouf, 72388, Saudi Arabia

[2] Department of Pharmaceutics, College of Pharmacy, Jouf University, Sakaka, Al Jouf, 72388, Saudi Arabia

[3] Center for Ultrasound Molecular Imaging and Therapeutics, Pittsburgh Heart, Lung, Blood and Vascular Medicine Institute, University of Pittsburgh, Pittsburgh, PA, USA

[4]Centre for organic chemistry, School of Chemistry, University of the Punjab, Lahore, 54590, Pakistan

[5]Chemistry Department, College of Science, Jouf University, Al Jouf, 72388, Sakaka, Saudi Arabia

[6]Department of Clinical Laboratory Sciences, College of Applied Medical Sciences, Sakaka, Jouf University, Al Jouf, 72388, Saudi Arabia

[7]Department of Clinical Pharmacy, College of Pharmacy, Jouf University, Sakaka 72388, Saudi Arabia

[8]University of Kragujevac, Faculty of Science, Radoja Domanovića 12, 34000 Kragujevac, Serbia

[9]University of Kragujevac, Institute for Information Technologies Kragujevac, Department of Science, Jovana Cvijića bb, 34000 Kragujevac, Serbia

Corresponding author's e-mail address: sbukhari@ju.edu.sa and nenad.jankovic@kg.ac.rs

Contents

1. Experimental data of **3a-i** 3

2. NMR spectra of **3a-i** 5

2.1. ^{1}H NMR spectrum of 5-(4'-hydroxy-3'-methoxy-5'-nitrobenzylidene)-2,2-dimethyl-1,3-dioxane-4,6-dione **3a** 5

2.2. ^{13}C NMR spectrum of 5-(4'-hydroxy-3'-methoxy-5'-nitrobenzylidene)-2,2-dimethyl-1,3-dioxane-4,6-dione **3a** 6

2.3. ^{1}H NMR spectrum of 2'-methoxy-4'-((2,2-dimethyl-4,6-dioxo-1,3-dioxan-5-ylidene)methyl)phenyl propionate **3b** 7

2.4. ^{13}C NMR spectrum of 2'-methoxy-4'-((2,2-dimethyl-4,6-dioxo-1,3-dioxan-5-ylidene)methyl)phenyl propionate **3b** 8

2.5. ^{1}H NMR spectrum of 2'-methoxy-4'-((2,2-dimethyl-4,6-dioxo-1,3-dioxan-5-ylidene)methyl)phenyl cyclopropanecarboxylate **3c** 9

2.6. ^{13}C NMR spectrum of 2'-methoxy-4'-((2,2-dimethyl-4,6-dioxo-1,3-dioxan-5-ylidene)methyl)phenyl cyclopropanecarboxylate **3c** 10

2.7. ^{1}H NMR spectrum of 2'-methoxy-4'-((2,2-dimethyl-4,6-dioxo-1,3-dioxan-5-ylidene)methyl)phenyl 4-methoxybenzoate **3d** 11

2.8. ^{13}C NMR spectrum of 2'-methoxy-4'-((2,2-dimethyl-4,6-dioxo-1,3-dioxan-5-ylidene)methyl)phenyl 4-methoxybenzoate **3d** 12

2.9. ^{1}H NMR spectrum of 5-(4-(2-(2-(2-hydroxyethoxy)ethoxy)ethoxy)-3-methoxybenzylidene)-2,2-dimethyl-1,3-dioxane-4,6-dione **3e** 13

2.10. ^{13}C NMR spectrum 5-(4'-(2''-(2'''-(2''''-hydroxyethoxy)ethoxy)ethoxy)-3'-methoxybenzylidene)-2,2-dimethyl-1,3-dioxane-4,6-dione **3e** 14

2.11. ^{1}H NMR spectrum of 5-(4'-hexyloxy-3'-methoxybenzylidene)-2,2-dimethyl-1,3-dioxane-4,6-dione **3f** 15

2.12. ^{13}C NMR spectrum of 5-(4'-hexyloxy-3'-methoxybenzylidene)-2,2-dimethyl-1,3-dioxane-4,6-dione **3f** 16

2.13. ^{1}H NMR spectrum of 5-(4'-heptyloxy-3'-methoxybenzylidene)-2,2-dimethyl-1,3-dioxane-4,6-dione **3g** 17

2.14. ^{13}C NMR spectrum of 5-(4'-heptyloxy-3'-methoxybenzylidene)-2,2-dimethyl-1,3-dioxane-4,6-dione **3g** 18

2.15. ^{1}H NMR spectrum of 5-(3'-methoxy-4'-octyloxybenzylidene)-2,2-dimethyl-1,3-dioxane-4,6-dione **3h** 19

2.16. ^{13}C NMR spectrum of 5-(3'-methoxy-4'-octyloxybenzylidene)-2,2-dimethyl-1,3-dioxane-4,6-dione **3h** 20

2.17. ^{1}H NMR spectrum of 5-(4'-decyloxy-3'-methoxybenzylidene)-2,2-dimethyl-1,3-dioxane-4,6-dione **3i** 21

2.18. ^{13}C NMR spectrum of 5-(4'-decyloxy-3'-methoxybenzylidene)-2,2-dimethyl-1,3-dioxane-4,6-dione **3i** 22

3. Molecular docking 23

1. Experimental data of 3a-i

5-(4’-hydroxy-3’-methoxy-5’-nitrobenzylidene)-2,2-dimethyl-1,3-dioxane-4,6-dione 3a

Green solid; Yield: 79%; IR ν 3204, 2949, 1751, 1703, 1577, 1541 cm^{-1}; ^1H NMR (200 MHz, DMSO-d_6) δ 8.54 (s, 1H), 8.33 (s, 1H), 8.14 (s, 1H), 3.92 (s, 3H), 1.74(s, 6H) ppm**;** ^{13}C NMR (50 MHz, DMSO-d_6) δ 162.8, 160.0, 155.1, 147.3, 124.2, 121.8, 120.2, 113.5, 104.5, 56.9, 27.2 ppm; ESI-MS (*m/z*): [M + Na] = 346.

2’-methoxy-4’-((2,2-dimethyl-4,6-dioxo-1,3-dioxan-5-ylidene)methyl)phenyl propionate 3b

Light green solid; Yield: 63%; IR ν 3465, 2991, 2944, 1762, 1733, 1598, 1582, 1512 cm^{-1}; ^1H NMR (200 MHz, $CDCl_3$) δ 8.36 (s, 1H), 8.22 (d, *J* = 2.0 Hz, 1H), 7.63 – 7.51 (m, 1H), 7.15 (d, *J* = 8.2 Hz, 1H), 3.90 (s, 3H), 2.65 (q, *J* = 7.5 Hz, 2H), 1.80 (s, 6H), 1.29 (t, *J* = 7.5 Hz, 3H) ppm; ^{13}C NMR (50 MHz, $CDCl_3$) δ 171.7, 163.3, 159.8, 157.1, 151.1, 144.6, 130.2, 129.1, 123.0, 116.9, 114.1, 104.5, 56.1, 27.6, 27.4, 9.1 ppm; ESI-MS (*m/z*): [M + H] = 335.

2’-methoxy-4’-((2,2-dimethyl-4,6-dioxo-1,3-dioxan-5-ylidene)methyl)phenyl cyclopropanecarboxylate 3c

Light green solid; Yield: 61%; IR ν 3469, 2985, 1753, 1733, 1513, 1379 cm^{-1}; ^1H NMR (200 MHz, $CDCl_3$) δ 8.36 (s, 1H), 8.22 (d, *J* = 2.0 Hz, 1H), 7.57 (dd, *J* = 8.4, 2.0 Hz, 1H), 7.17 (d, *J* = 8.3 Hz, 1H), 3.91 (s, 3H), 1.96 – 1.80 (m, 7H), 1.23 – 1.01 (m, 4H) ppm; ^{13}C NMR (50 MHz, $CDCl_3$) δ 172.1, 163.3, 159.8, 157.1, 151.2, 144.6, 130.2, 129.1, 123.1, 116.9, 114.1, 104.46, 56.1, 27.6, 12.9, 9.5 ppm; ESI-MS (*m/z*): [M + H] = 347.

2’-methoxy-4’-((2,2-dimethyl-4,6-dioxo-1,3-dioxan-5-ylidene)methyl)phenyl 4-methoxybenzoate 3d

Green solid; Yield: 69%; IR ν 3446, 2993, 1732, 1606, 1577 cm^{-1}; ^1H NMR (200 MHz, $CDCl_3$) δ 8.40 (s, 1H), 8.26 (d, *J* = 1.8 Hz, 1H), 8.15 (t, *J* = 5.7 Hz, 2H), 7.62 (dd, *J* = 8.3, 1.9 Hz, 1H), 7.30 – 7.26 (m, 1H), 6.98 (t, *J* = 5.7 Hz, 2H), 3.89 (d, *J* = 1.9 Hz, 6H), 1.81 (s, 6H) ppm; ^{13}C NMR (50 MHz, $CDCl_3$) δ 164.0, 163.7, 163.3, 159.8, 157.2, 151.4, 144.9, 132.5, 130.2, 129.2, 123.2, 121.1, 117.0, 114.1, 113.9, 104.5, 56.1, 55.5, 27.6 ppm; ESI-MS (*m/z*): [M + H] = 413.

5-(4-(2-(2-(2-hydroxyethoxy)ethoxy)ethoxy)-3-methoxybenzylidene)-2,2-dimethyl-1,3-dioxane-4,6-dione 3e

Green solid; Yield: 54%; IR ν 3583, 3439, 2939, 2913, 1745, 1708, 1578, 1559 cm^{-1}; ^1H NMR (200 MHz, $CDCl_3$) δ 8.35 (s, 1H), 8.28 (d, *J* = 2.0 Hz, 1H), 7.63 (dd, *J* = 8.6, 2.0 Hz, 1H), 6.97 (d, *J* = 8.5 Hz, 1H), 4.33 – 4.25 (m, 2H), 3.95-3.85 (m, 5H), 3.76-3.59 (m, 8H), 2.58 (s, 1H), 1.80 (s, 6H) ppm; ^{13}C NMR (50 MHz, $CDCl_3$) δ 163.9, 160.4, 158.0, 153.9, 148.9, 132.2, 125.2, 116.2, 111.9, 110.7, 104.1, 72.5, 70.9, 70.3, 69.2, 68.5, 61.7, 55.9, 27.5 ppm; ESI-MS (*m/z*): [M + H] = 411.

5-(4’-hexyloxy-3’-methoxybenzylidene)-2,2-dimethyl-1,3-dioxane-4,6-dione 3f

Yellow solid; Yield: 75%; IR ν 3444, 2956, 2937, 1748, 1713, 1558, 1523 cm^{-1}; ^1H NMR (200 MHz, $CDCl_3$) δ 8.36 (s, 1H), 8.29 (d, *J* = 2.1 Hz, 1H), 7.64 (dd, *J* = 8.6, 2.1 Hz, 1H), 6.94 (d, *J* = 8.6 Hz, 1H), 4.13 (t, *J* = 6.9 Hz, 2H), 3.94 (s, 3H), 1.93 – 1.82 (m, 8H), 1.56 – 1.26 (m, 6H), 0.94-0.87 (m, 3H) ppm; ^{13}C NMR (50 MHz, $CDCl_3$) δ 164.1, 160.5, 158.2, 154.5, 148.9, 132.6, 124.7, 116.0, 111.4, 110.2, 104.0, 69.2, 56.0, 31.5, 28.8, 27.5, 25.5, 22.5, 14.0 ppm; ESI-MS (*m/z*): [M + H] = 363.

5-(4’-heptyloxy-3’-methoxybenzylidene)-2,2-dimethyl-1,3-dioxane-4,6-dione 3g

Yellow solid; Yield: 92%; IR ν 3436, 2949, 2923, 1746, 1708, 1548, 1522 cm^{-1}; ^1H NMR (200 MHz, $CDCl_3$) δ 8.36 (s, 1H), 8.29 (d, *J* = 2.1 Hz, 1H), 7.64 (dd, *J* = 8.6, 2.1 Hz, 1H), 6.94 (d, *J* = 8.5 Hz, 1H), 4.13 (t, *J* = 6.9 Hz, 2H), 3.94 (s, 3H), 2.06 – 1.77 (m, 8H), 1.77 – 1.03 (m, 8H), 1.03 – 0.79 (m, 3H) ppm; ^{13}C NMR (50 MHz, $CDCl_3$) δ 164.1, 160.5, 158.2, 154.5, 148.9, 132.6, 124.7, 116.1, 111.4, 110.3, 104.0, 69.2, 56.0, 31.7, 29.0, 28.9, 27.5, 25.8, 22.6, 14.1 ppm; ESI-MS (*m/z*): [M + H] = 377.

5-(3’-methoxy-4’-octyloxybenzylidene)-2,2-dimethyl-1,3-dioxane-4,6-dione 3h

Yellow solid; Yield: 88%; IR ν 3439, 2950, 2930, 1745, 1708, 1549, 1523 cm^{-1}; ^1H NMR (200 MHz, $CDCl_3$) δ 8.36 (s, 1H), 8.30 (d, *J* = 2.1 Hz, 1H), 7.63 (d, *J* = 8.5 Hz, 1H), 6.94 (d, *J* = 8.6 Hz, 1H), 4.13 (t, *J* = 6.8 Hz, 2H), 3.94 (s, 3H), 2.05 – 1.62 (m, 8H), 1.60 – 1.16 (m, 10H), 0.89 (m, 3H) ppm; ^{13}C NMR (50 MHz, $CDCl_3$) δ 164.0, 160.5, 158.1, 154.5, 148.9, 132.5, 124.7, 116.1, 111.4, 110.3, 103.9, 69.2, 56.0, 31.7, 29.2, 29.1, 28.8, 27.5, 25.8, 22.6, 14.0 ppm; ESI-MS (*m*/*z*): [M + H] = 391.

5-(4’-decyloxy-3’-methoxybenzylidene)-2,2-dimethyl-1,3-dioxane-4,6-dione 3i

Yellow solid; Yield: 82%; IR ν 3439, 2952, 2928, 1746, 1709, 1545, 1521 cm^{-1}; ^1H NMR (200 MHz, $CDCl_3$) δ 8.36 (s, 1H), 8.30 (d, *J* = 2.1 Hz, 1H), 7.64 (dd, *J* = 8.6, 2.1 Hz, 1H), 6.94 (d, *J* = 8.6 Hz, 1H), 4.13 (t, *J* = 6.8 Hz, 2H), 3.94 (s, 3H), 2.00 – 1.68 (m, 8H), 1.62 – 1.16 (m, 14H), 0.88 (t, *J* = 6.4 Hz, 3H) ppm; ^{13}C NMR (50 MHz, $CDCl_3$) δ 164.0, 160.5, 158.1, 154.5, 148.9, 132.5, 124.7, 116.0, 111.4, 110.3, 104.0, 69.2, 56.0, 31.9, 29.5, 29.3, 29.3, 28.8, 27.5, 25.8, 22.6, 14.1 ppm; ESI-MS (*m*/*z*): [M + H] = 419.

2. NMR spectra of 3a-i

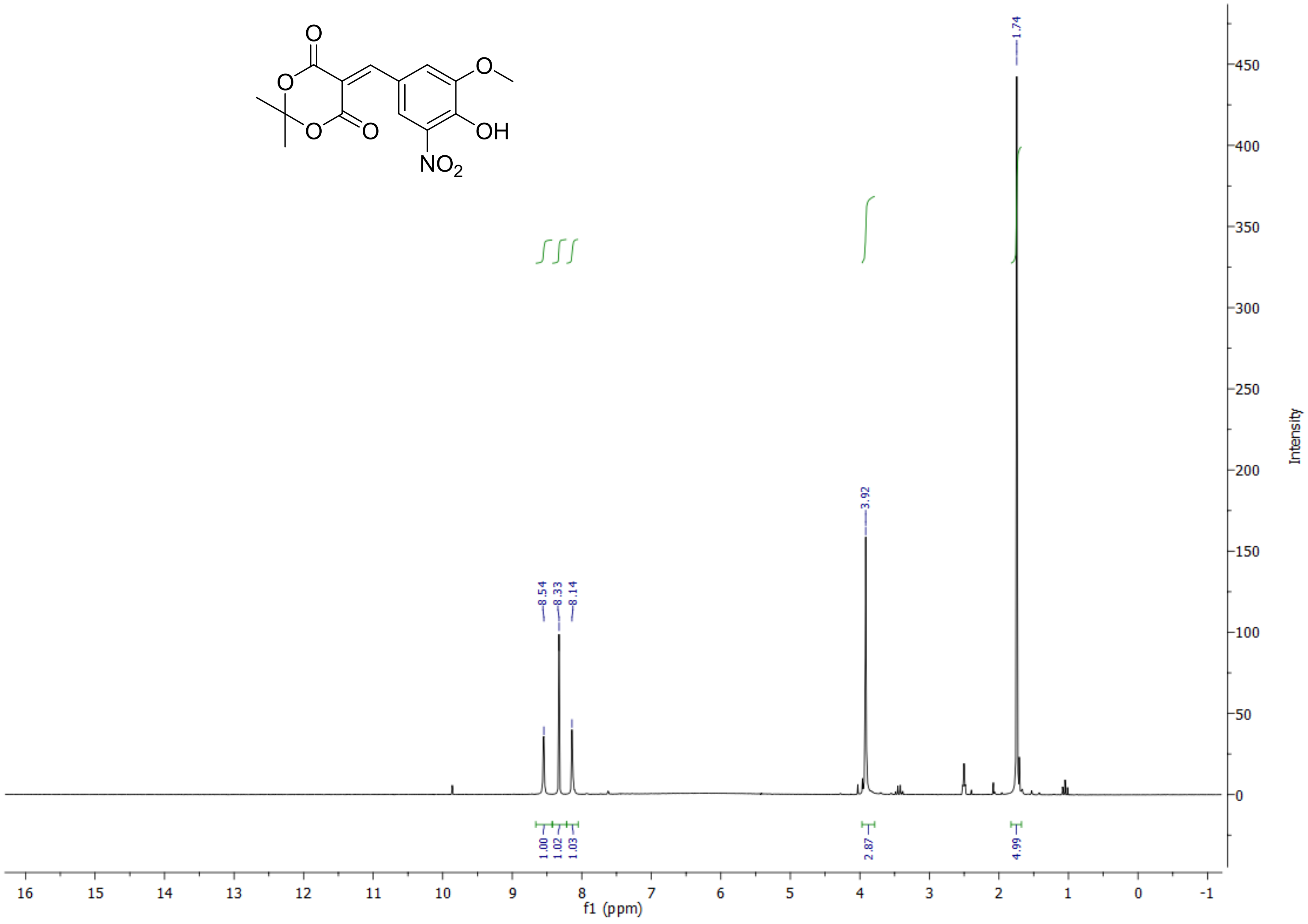

Figure S1. ^{1}H NMR spectrum (200 MHz) of **3a** (triplet at 1.1 ppm, and quartet at 3.45 are origin from solvent-ethanol).

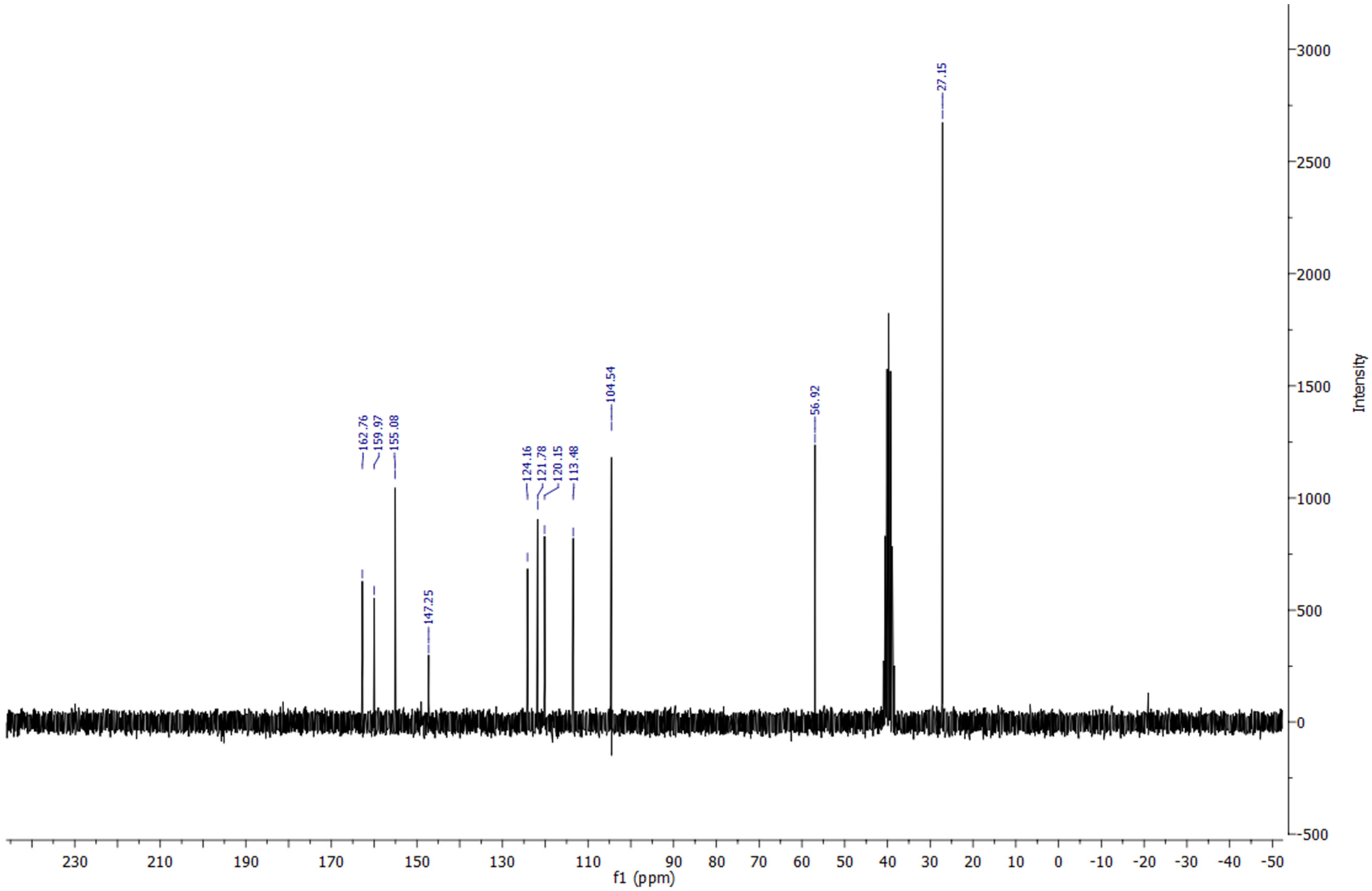

Figure S2. ^{13}C NMR spectrum (50 MHz) of **3a**.

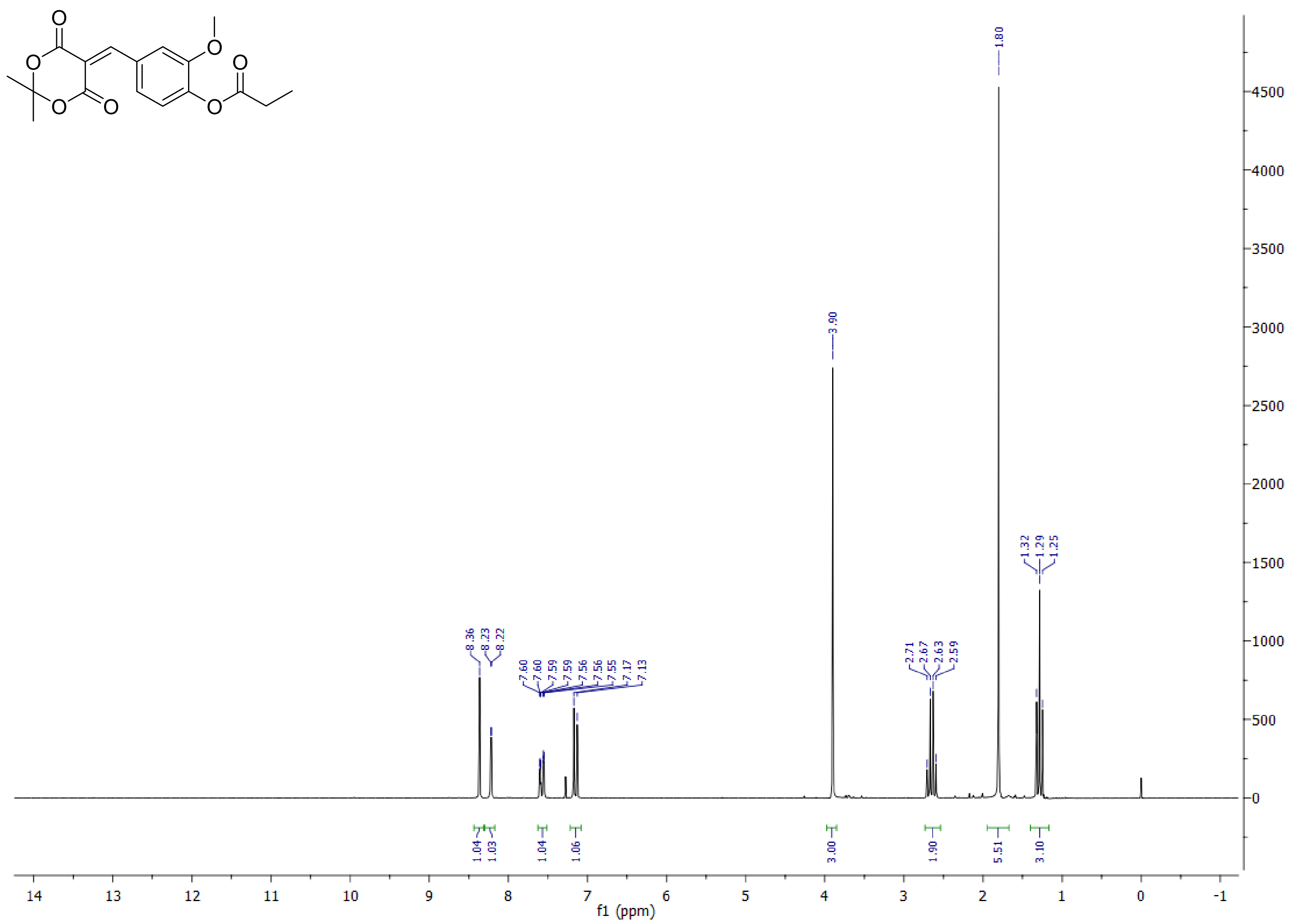

Figure S3. ^{1}H NMR spectrum (200 MHz) of **3b**.

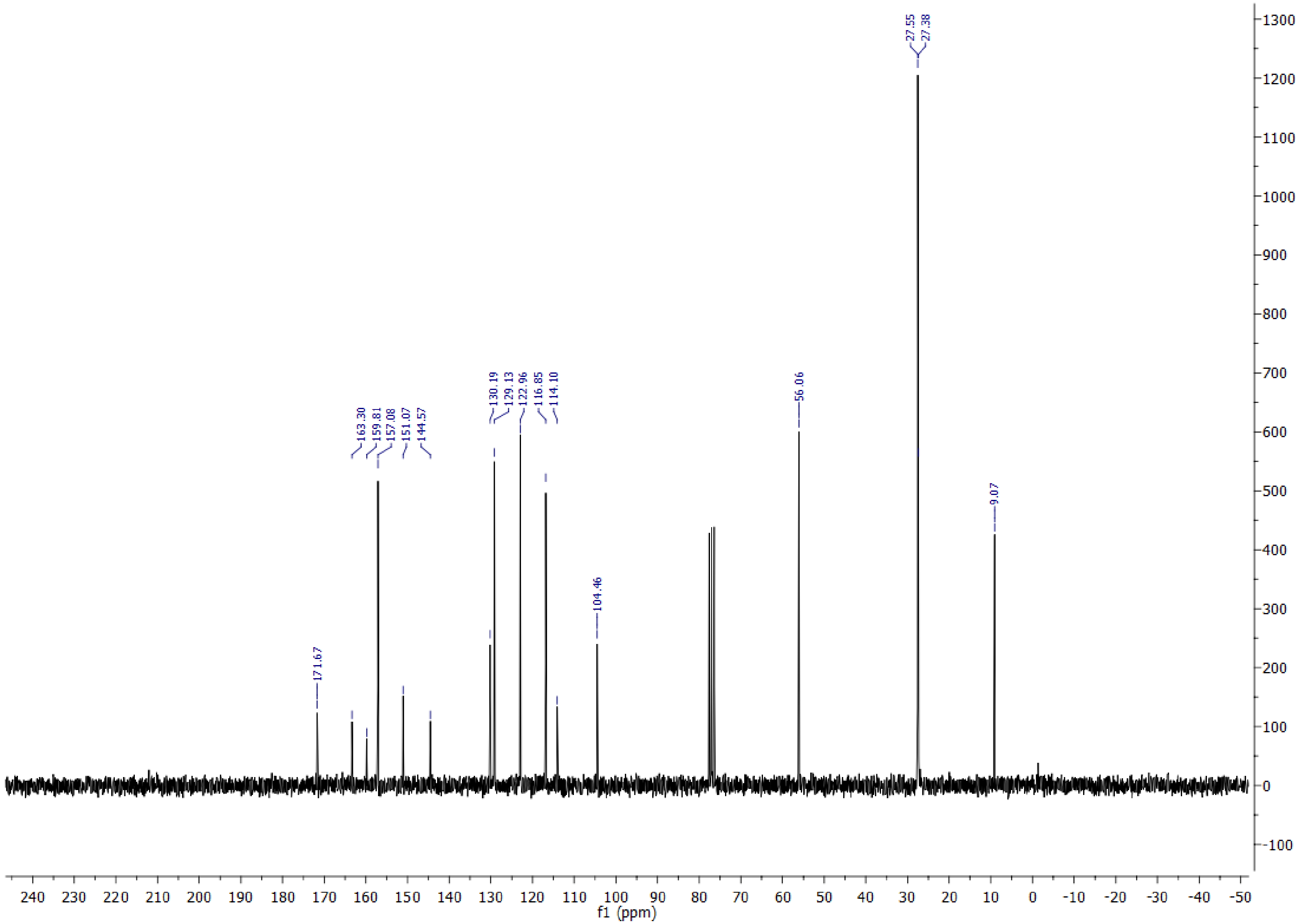

Figure S4. ^{13}C NMR spectrum (50 MHz) of **3b**.

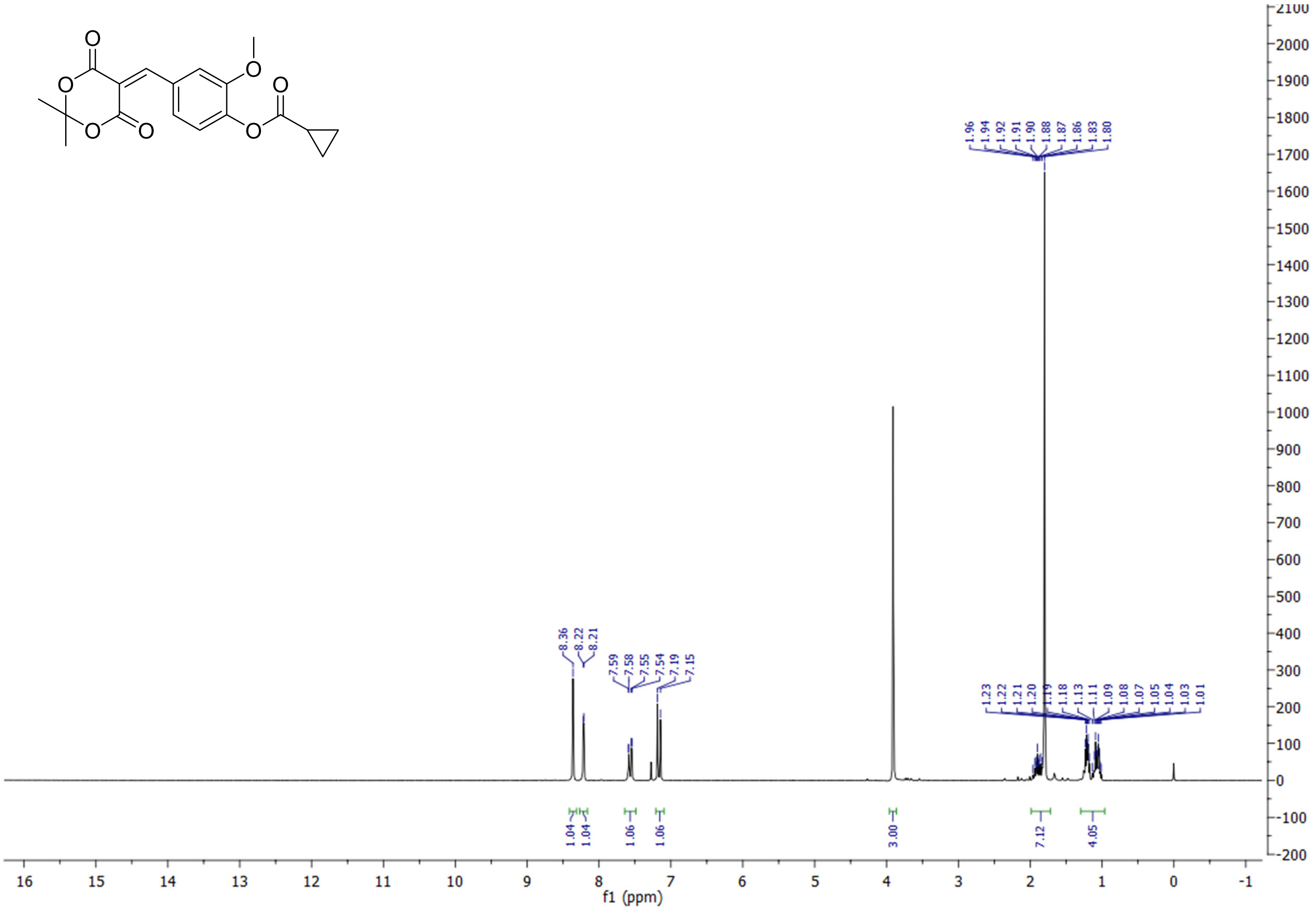

Figure S5. ^{1}H NMR spectrum (200 MHz) of **3c**.

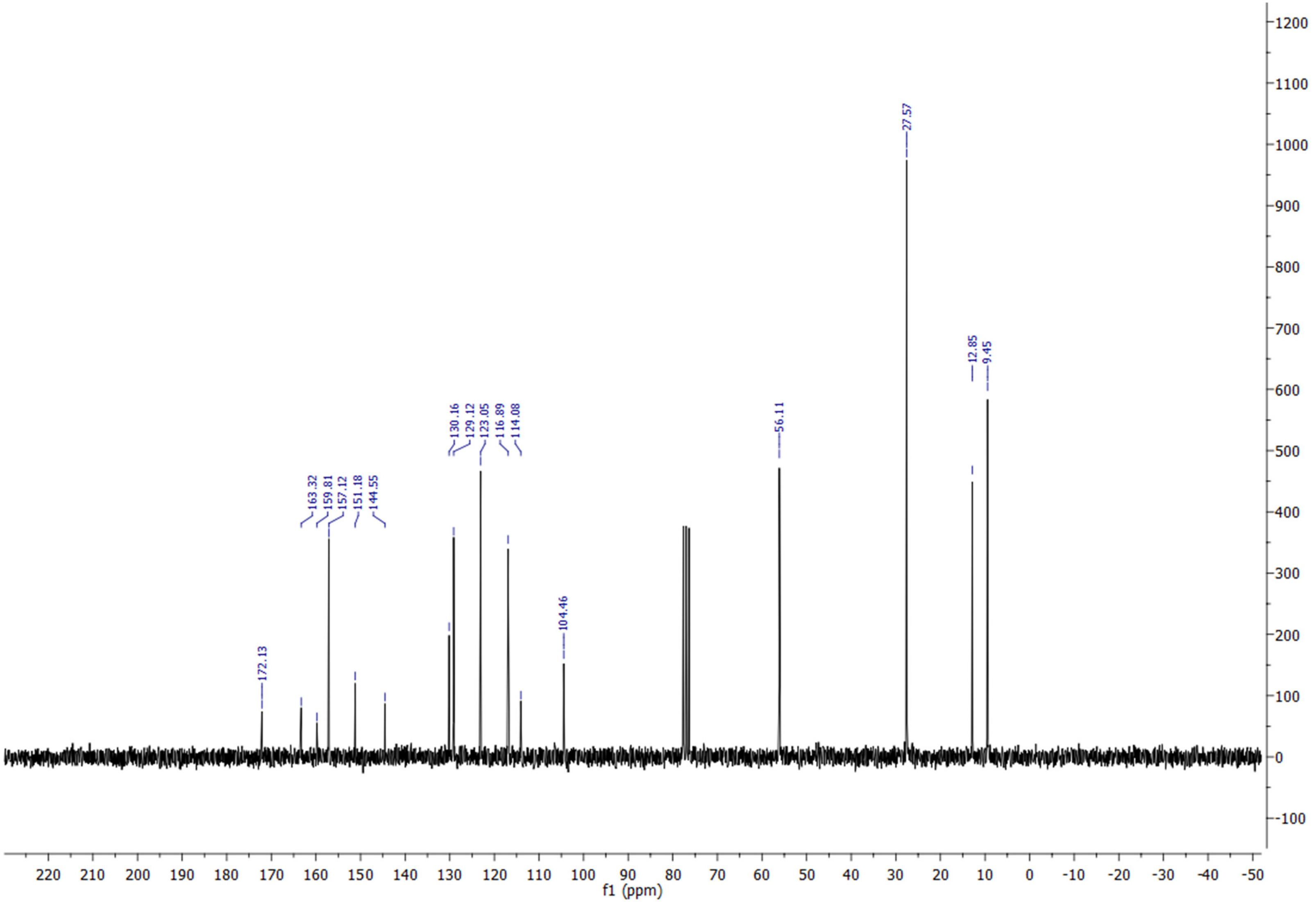

Figure S6. ^{13}C NMR spectrum (50 MHz) of **3c**.

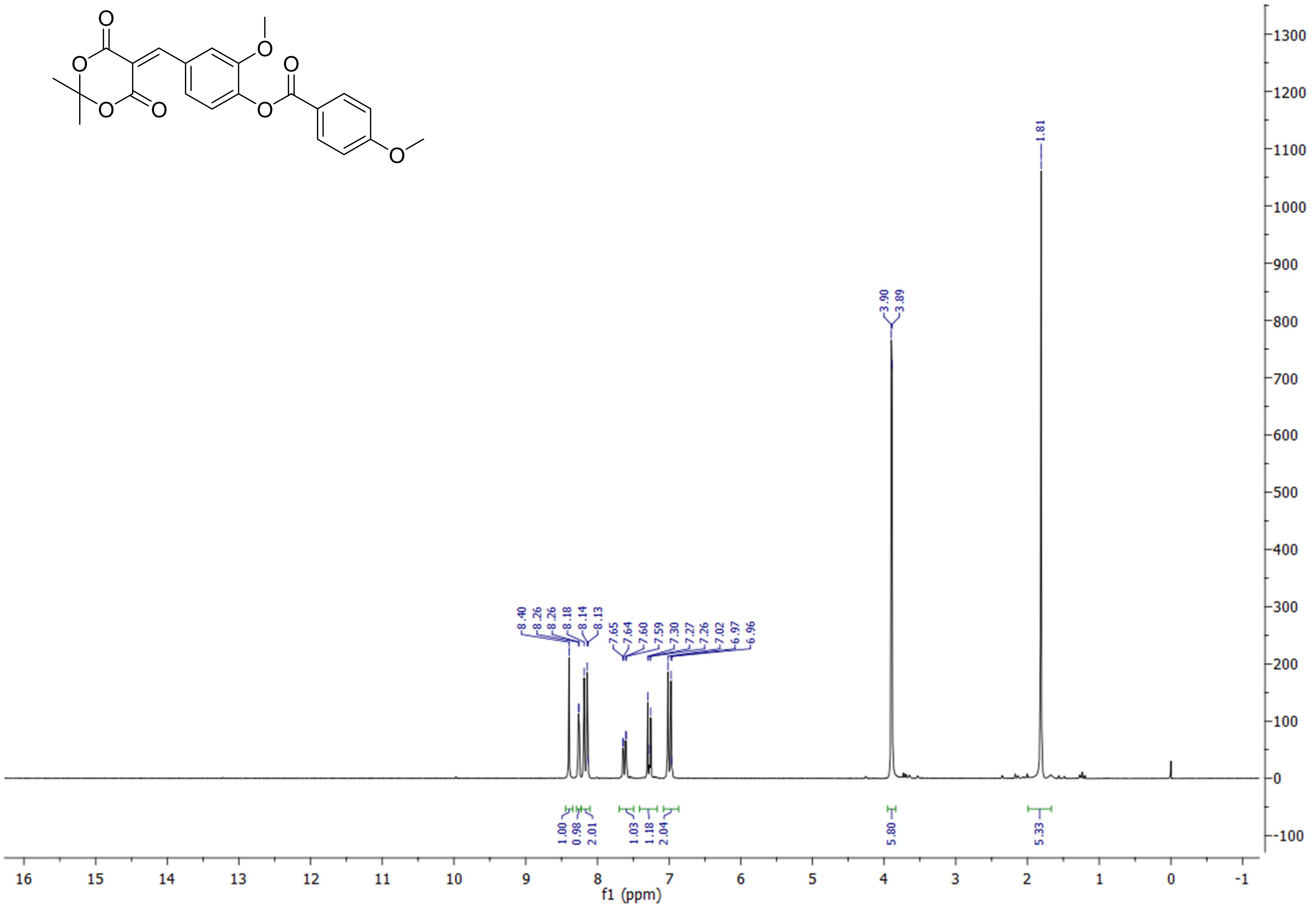

Figure S7. ^{1}H NMR spectrum (200 MHz) of **3d**.

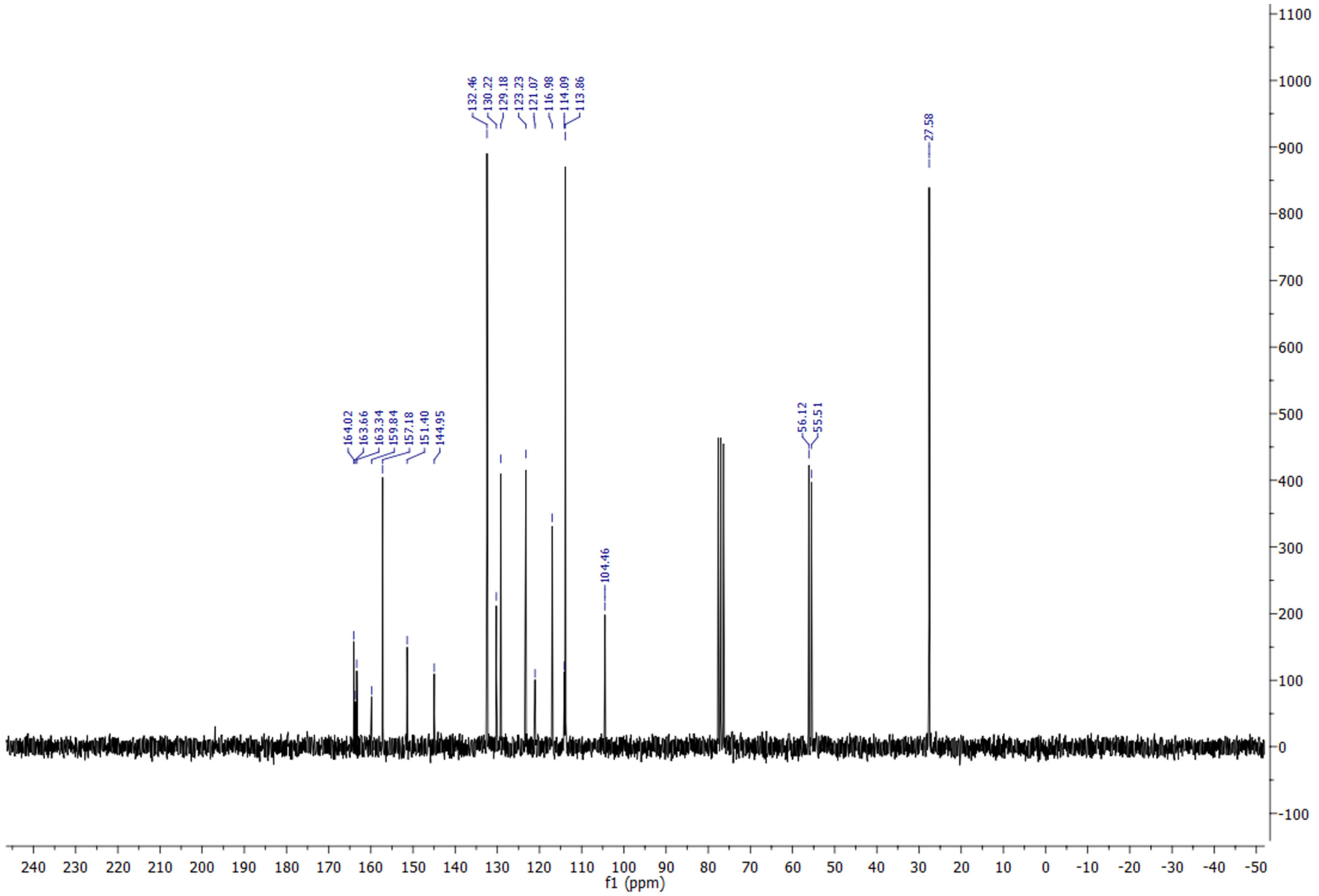

Figure S8. ^{13}C NMR spectrum (50 MHz) of **3d**.

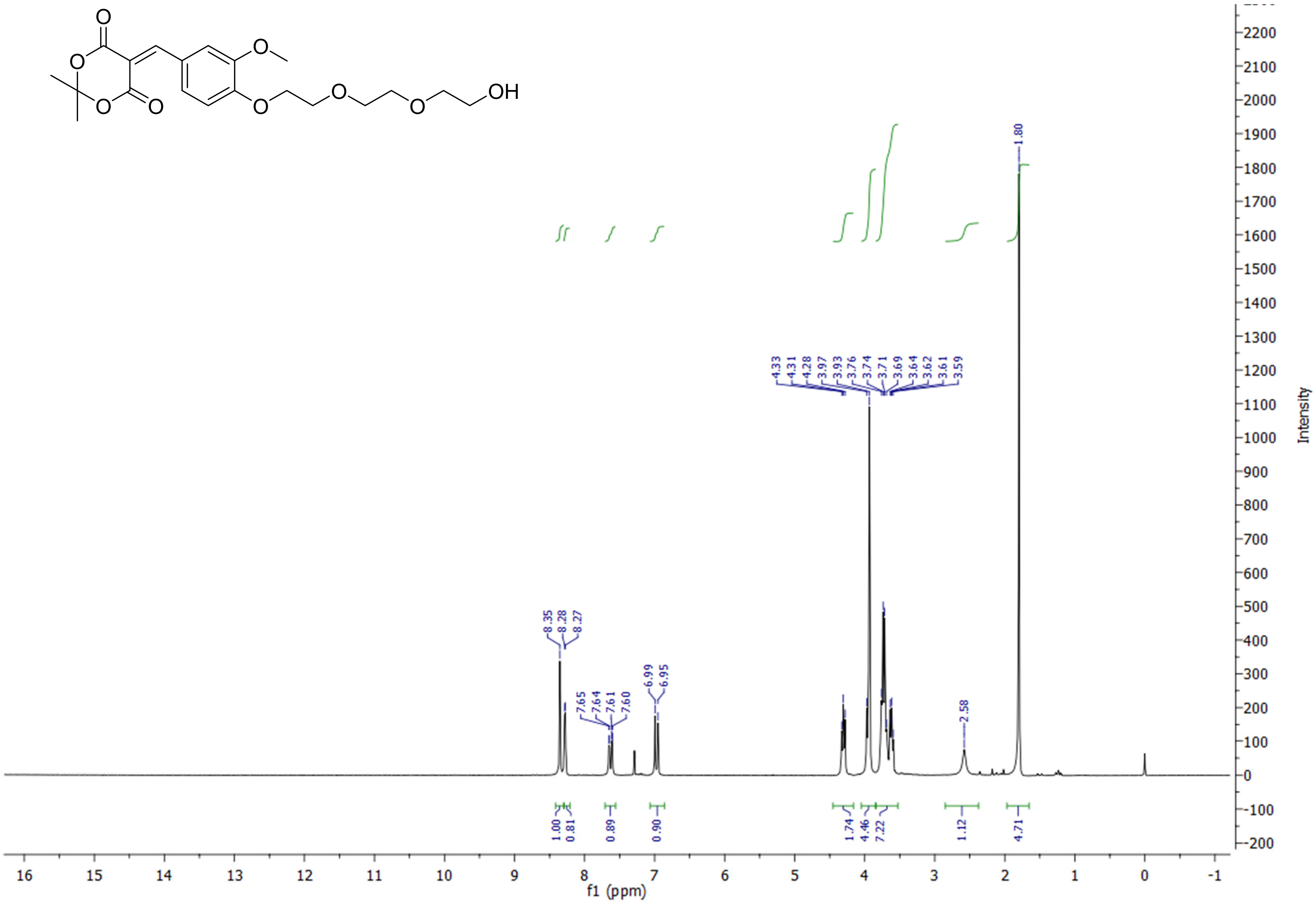

Figure S9. ^{1}H NMR spectrum (200 MHz) of **3e**.

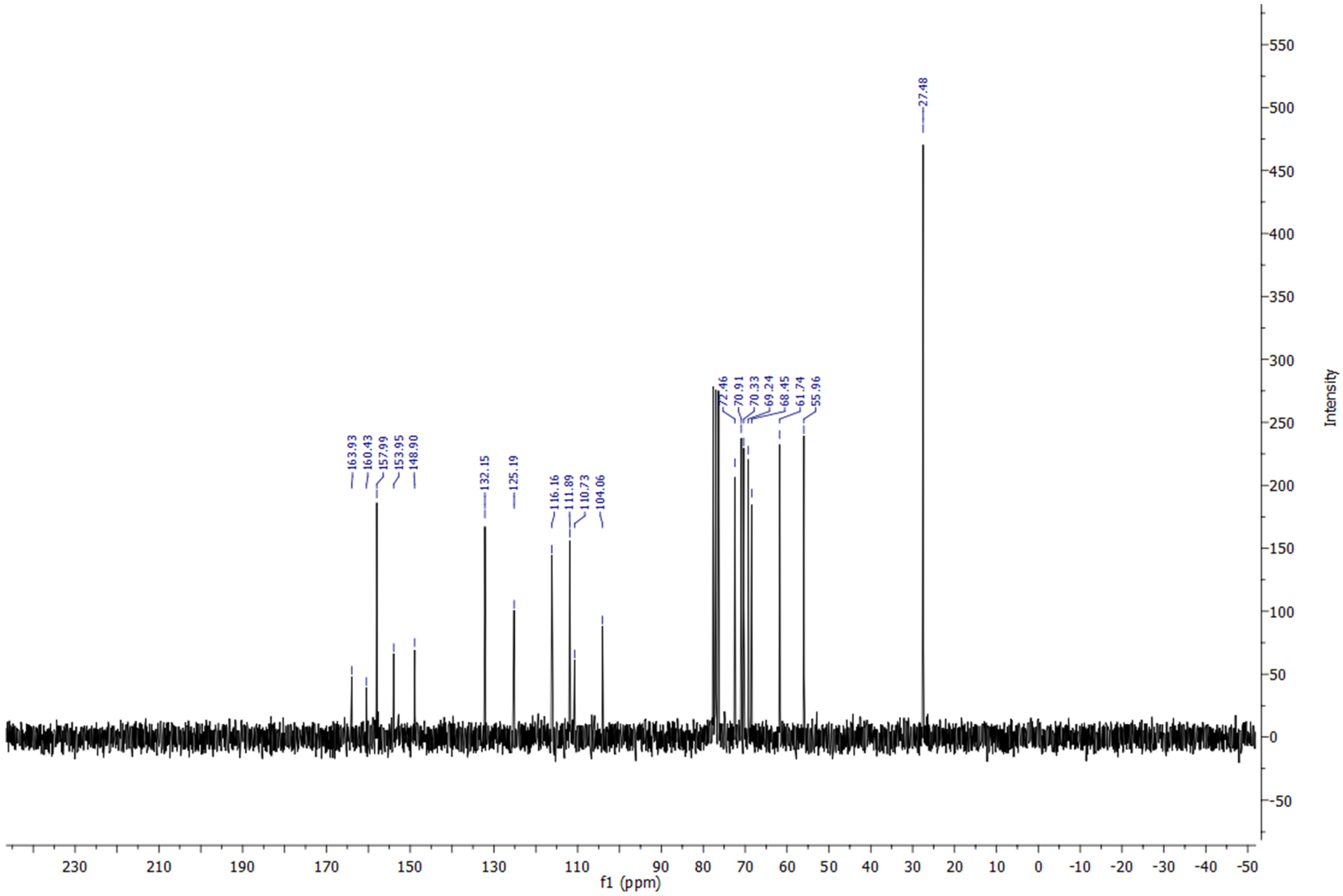

Figure S10. ^{13}C NMR spectrum (50 MHz) of **3e**.

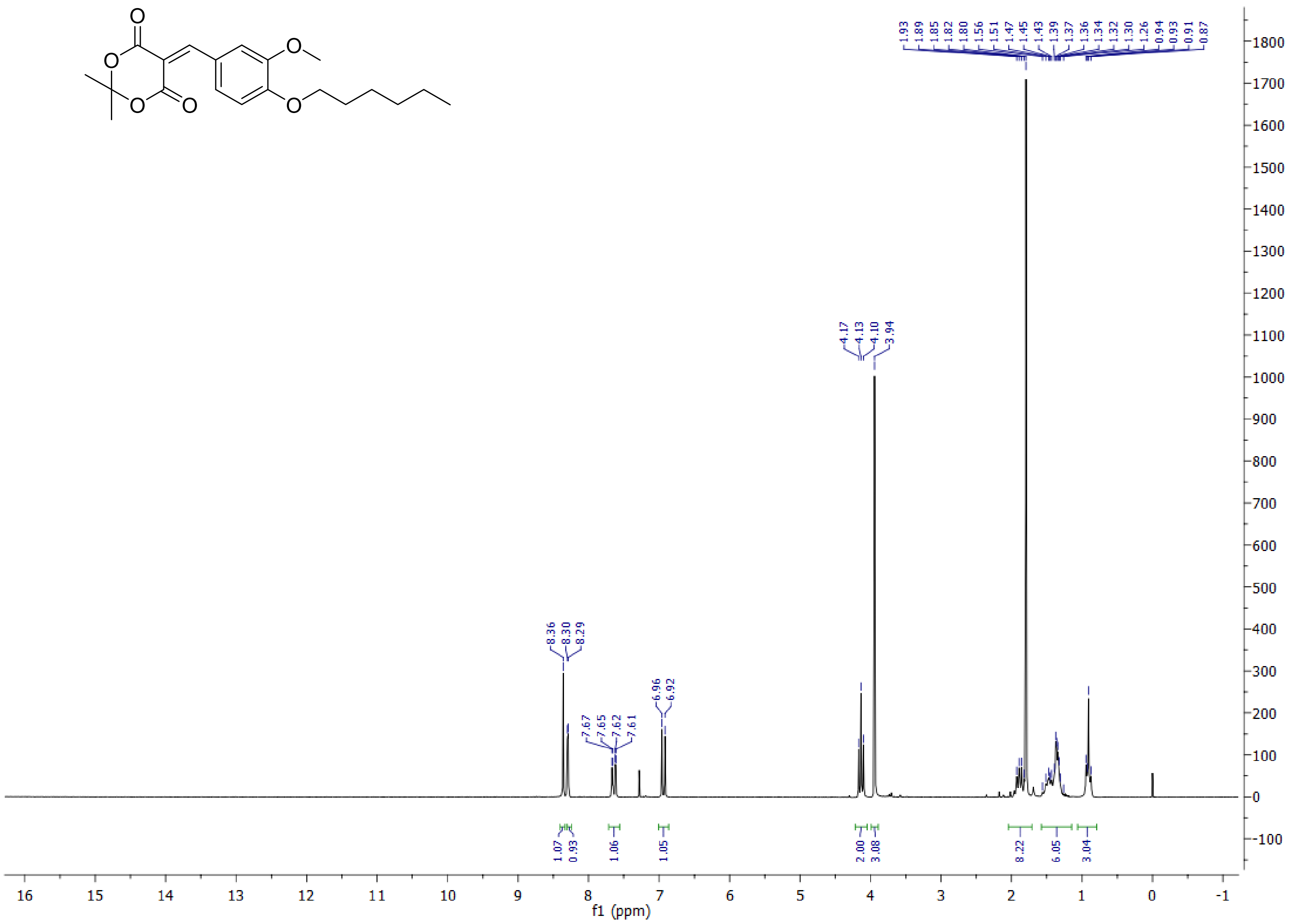

Figure S11. ^{1}H NMR spectrum (200 MHz) of **3f**.

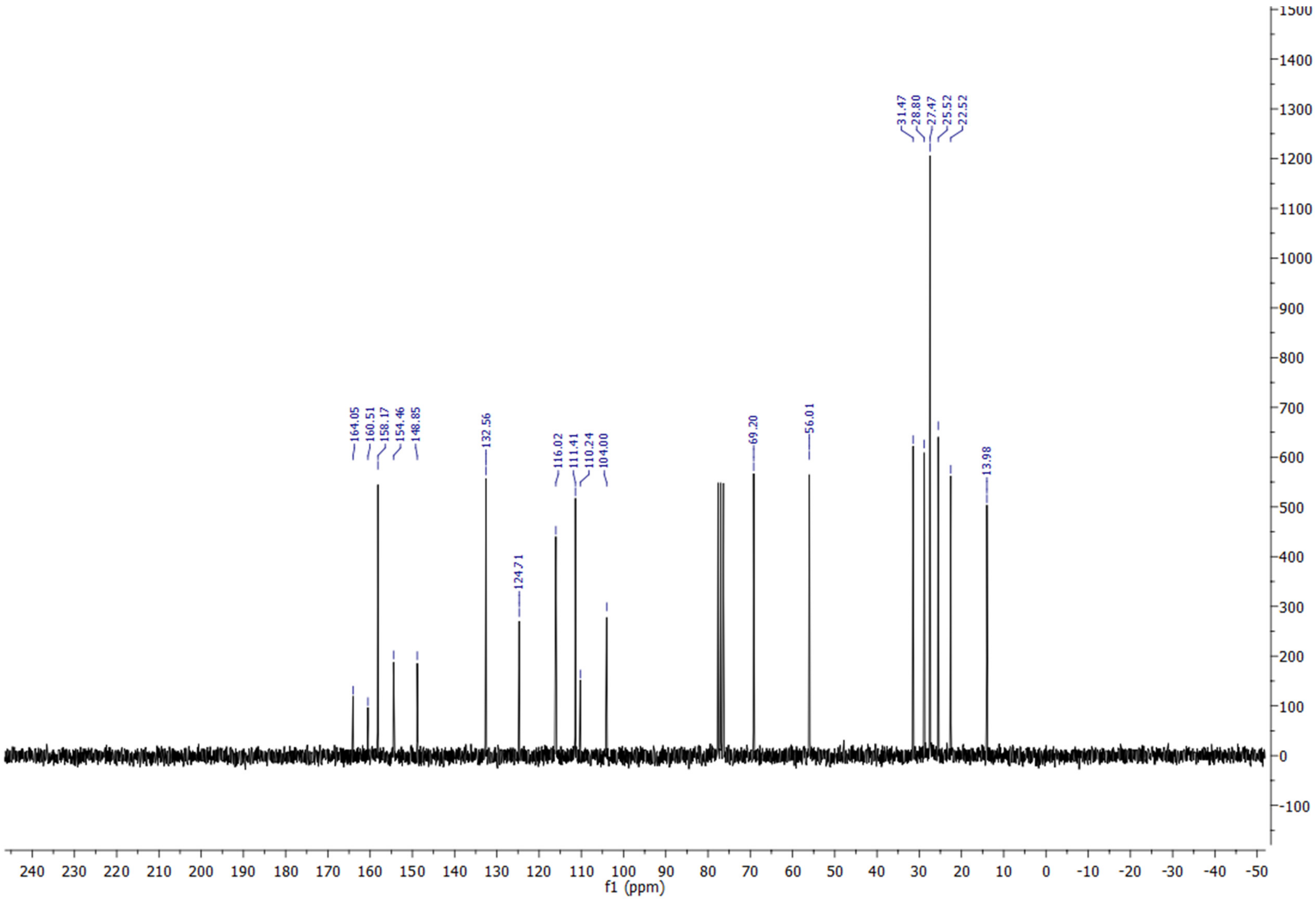

Figure S12. ^{13}C NMR spectrum (50 MHz) of **3f**.

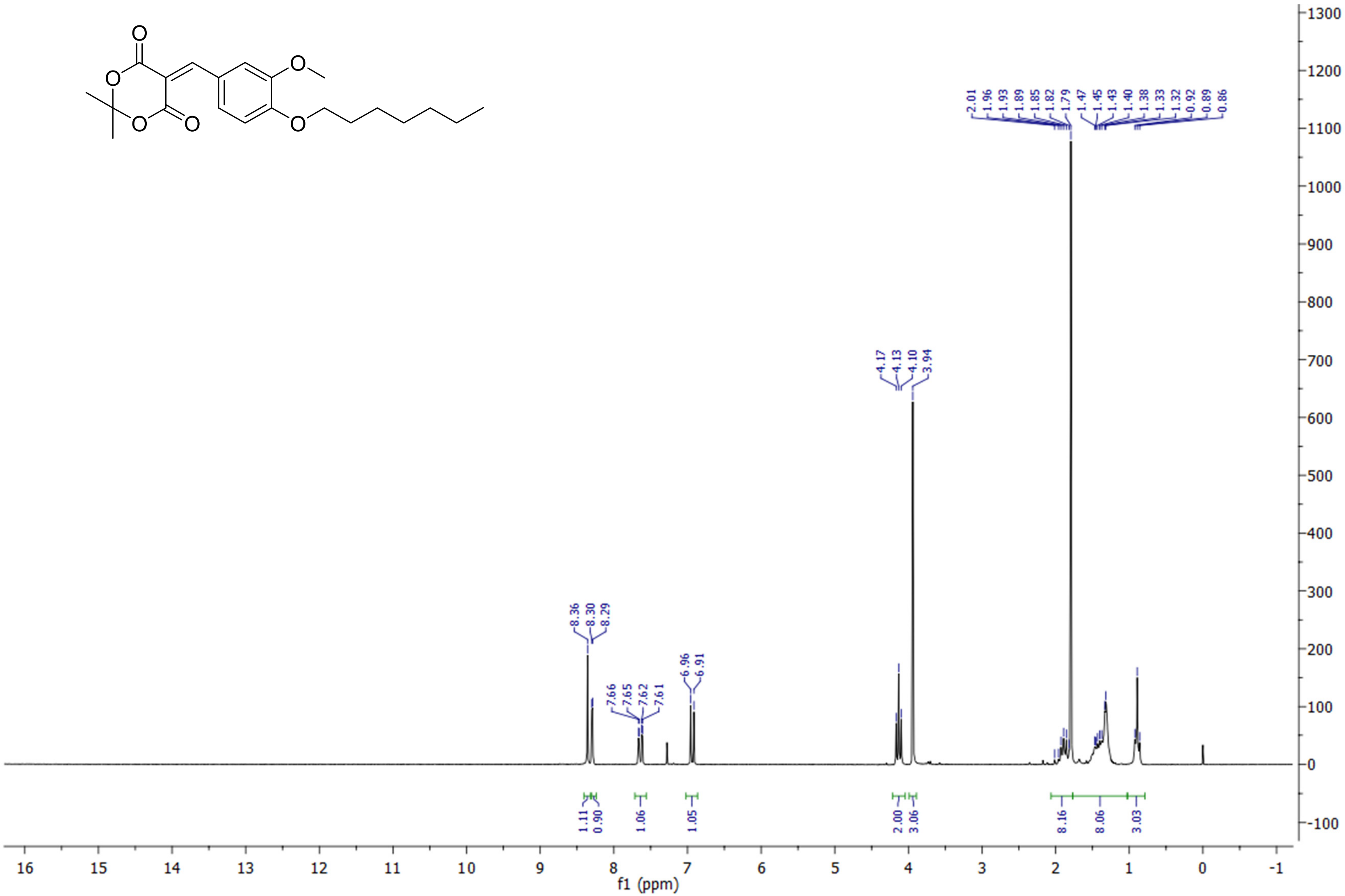

Figure S13. ^{1}H NMR spectrum (200 MHz) of **3g**.

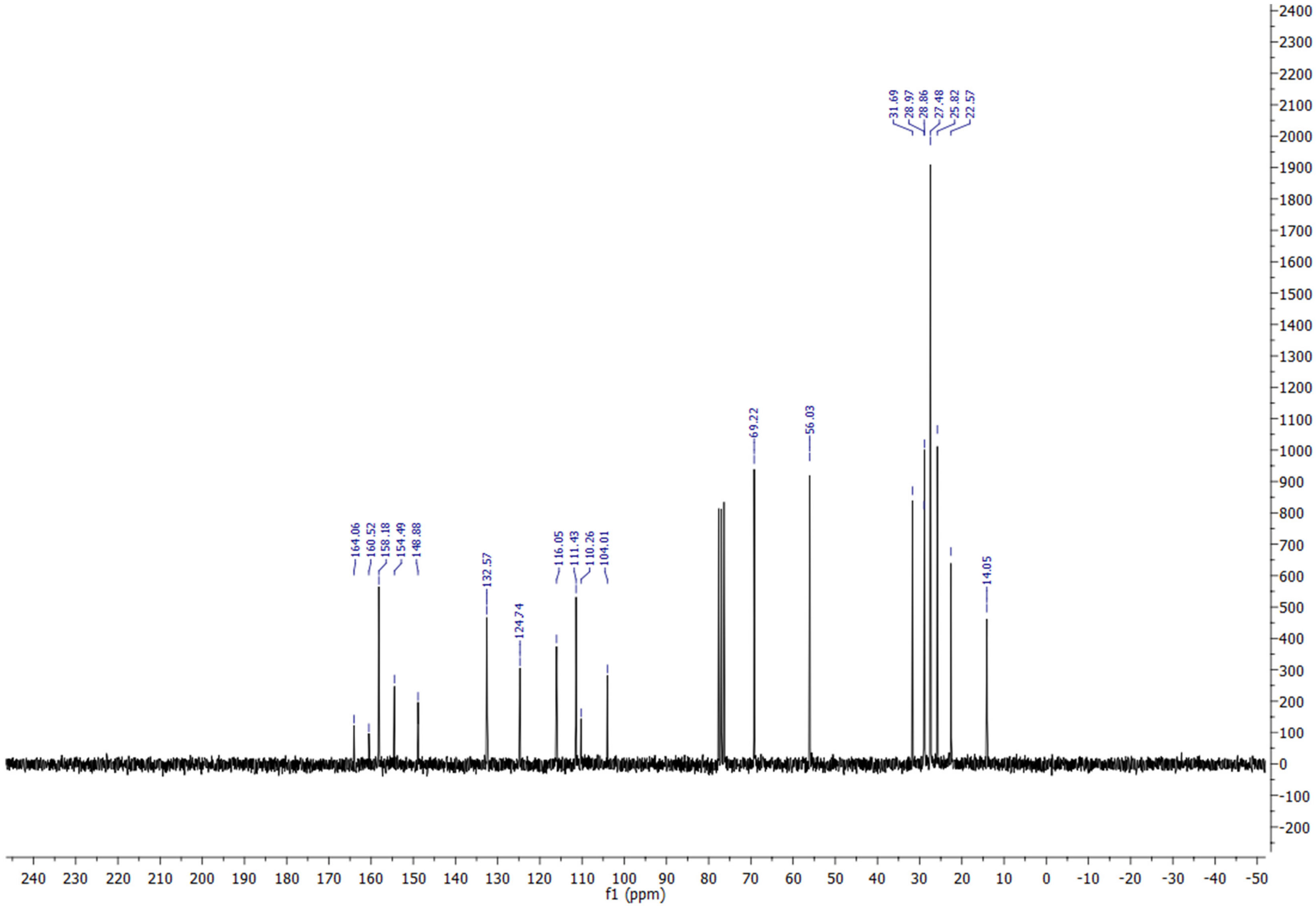

Figure S14. ^{13}C NMR spectrum (50 MHz) of **3g**.

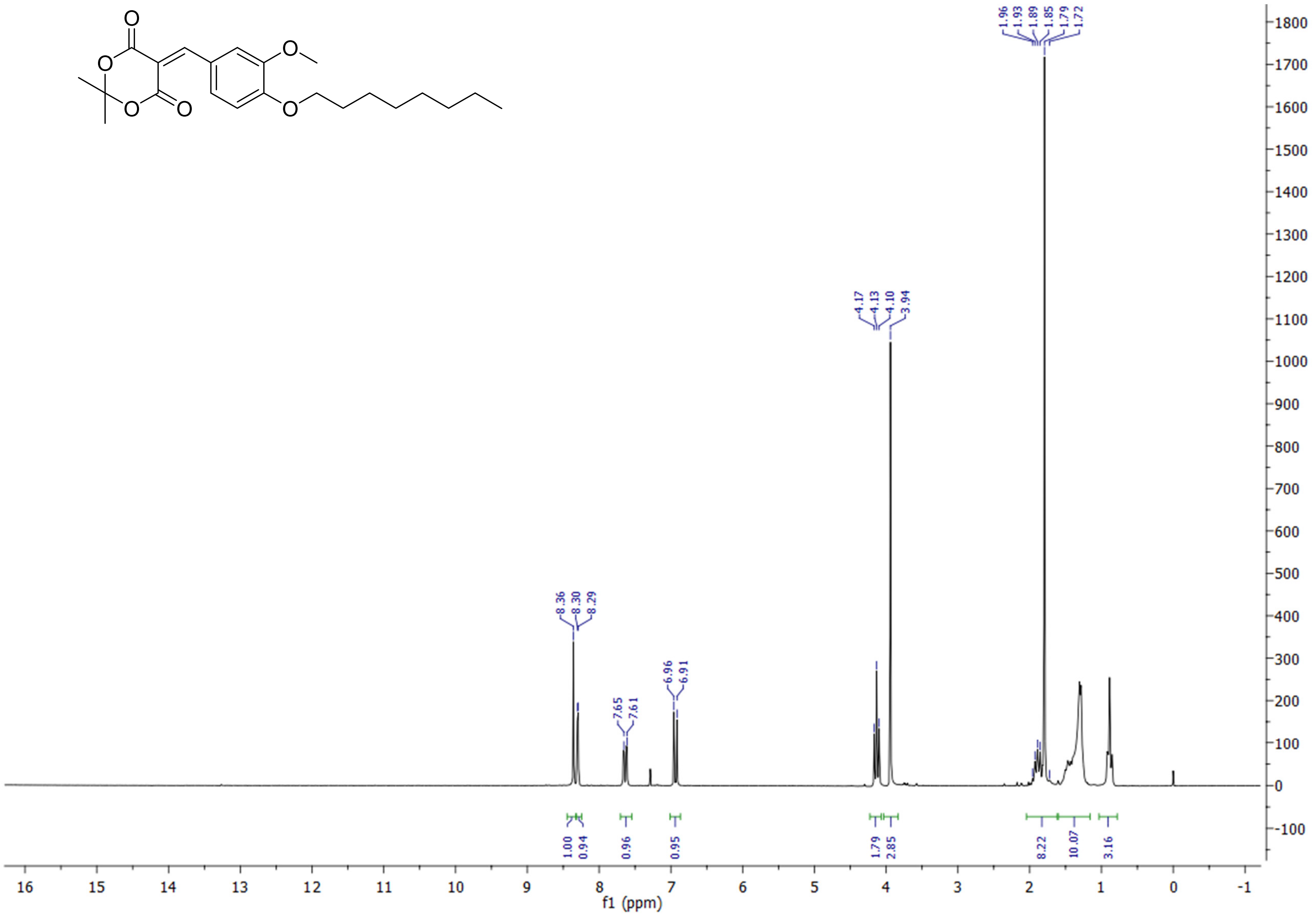

Figure S15. ^{1}H NMR spectrum (200 MHz) of **3h**.

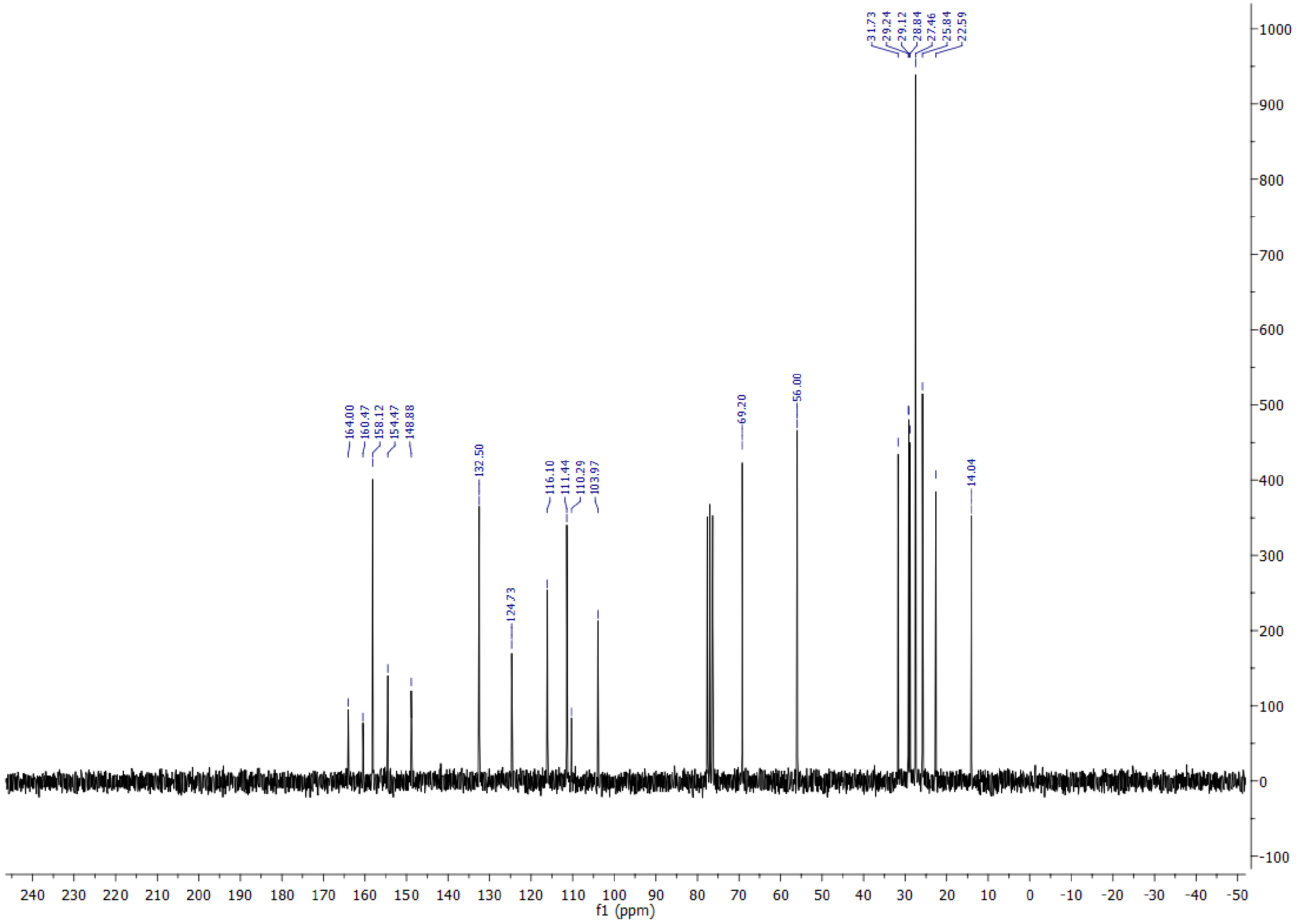

Figure S16. ^{13}C NMR spectrum (50 MHz) of **3h**.

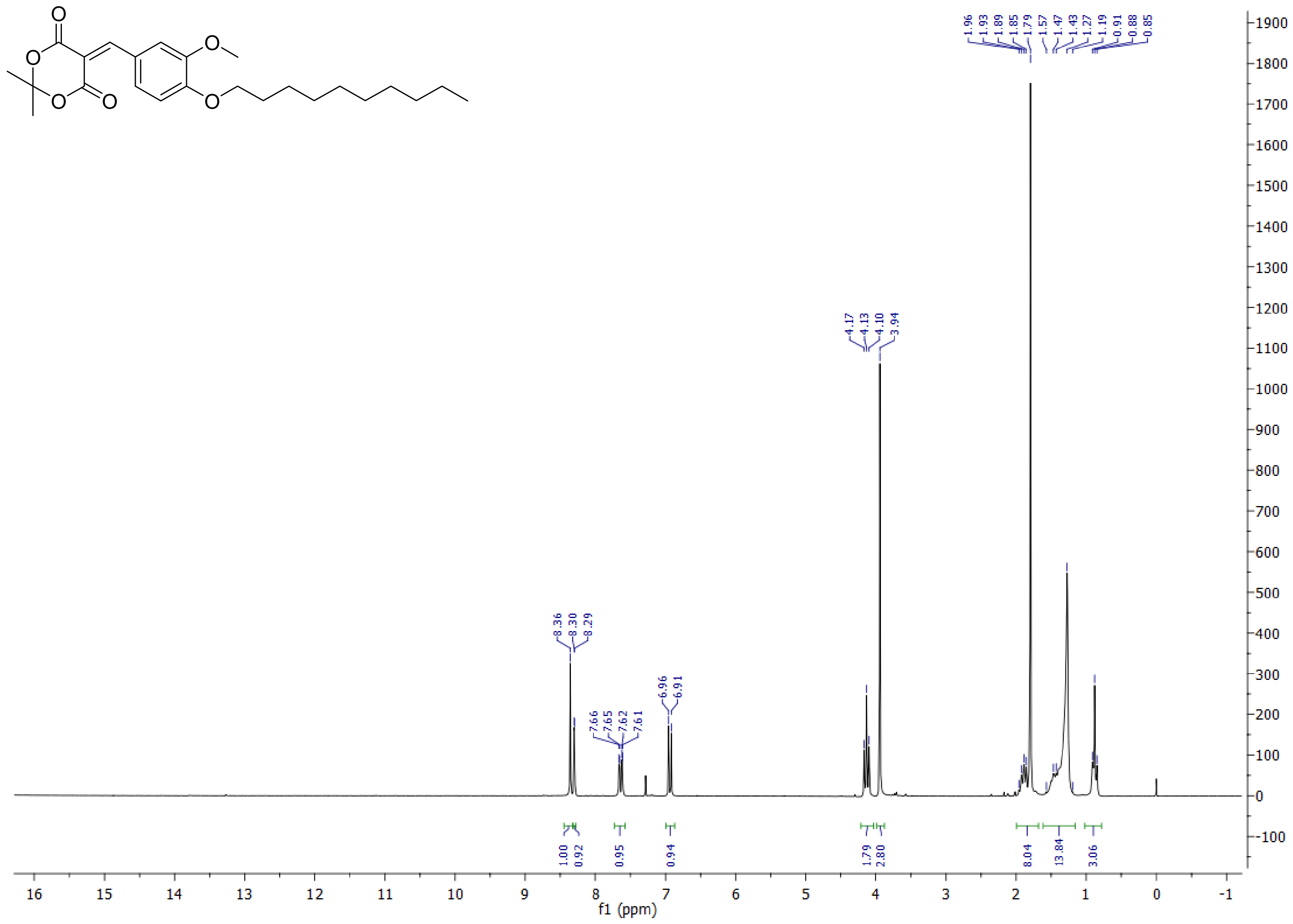

Figure S17. ^{1}H NMR spectrum (200 MHz) of **3i**.

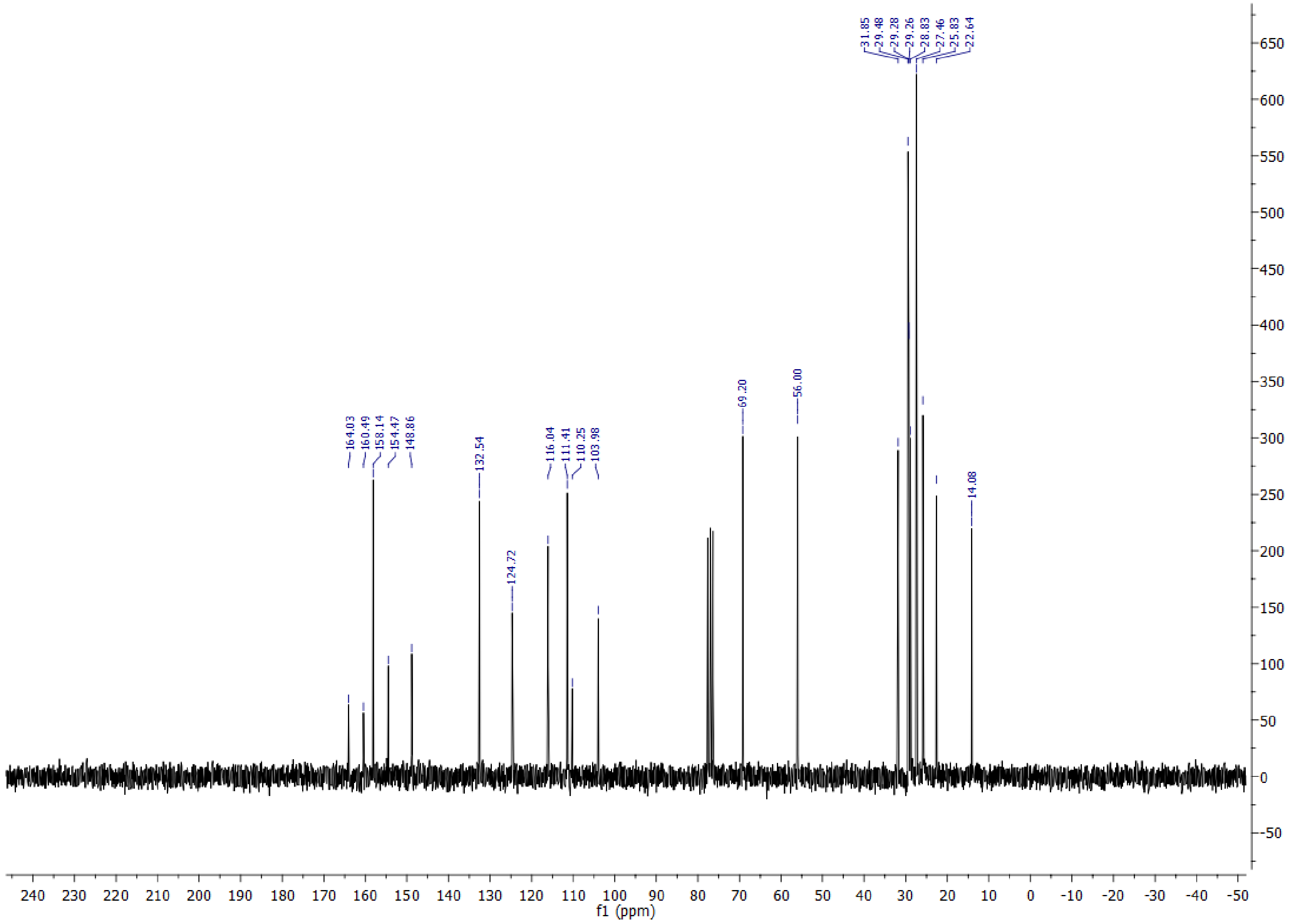

Figure S18. ^{13}C NMR spectrum (50 MHz) of **3i**.

3. Molecular docking

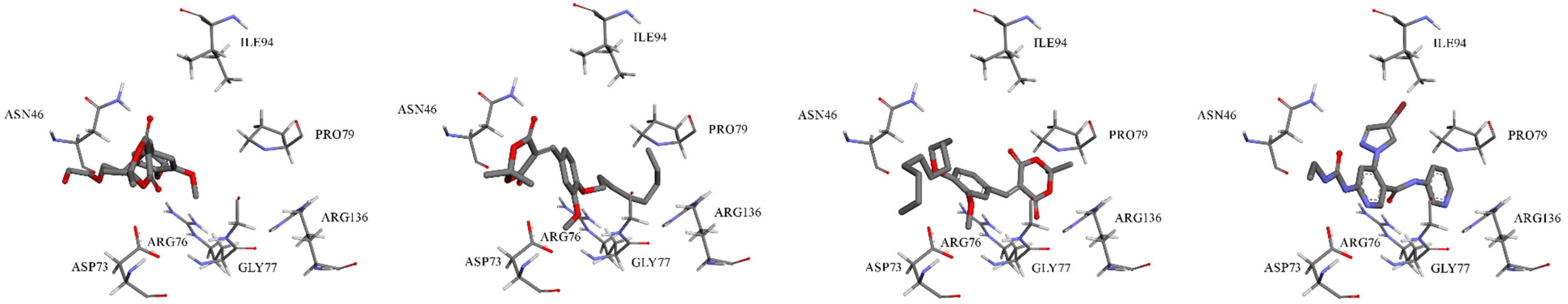

Figure S19. The lowest energy structures that resulted from docking of **3e**, **3h**, **3i**, and P3C (from left to right) to active site of Gyrase B of *E. coli.*

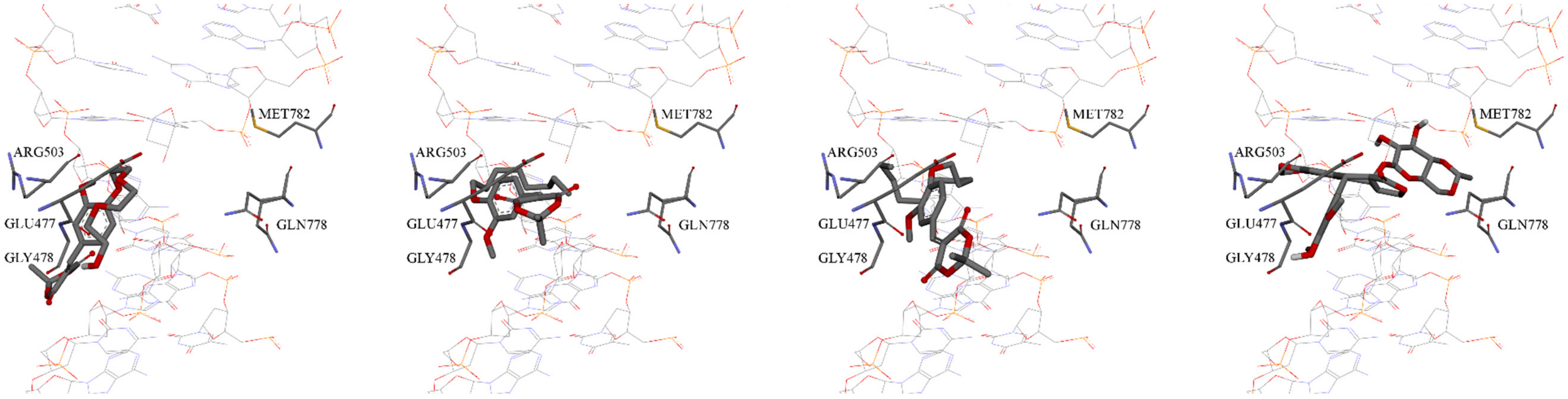

Figure S20. Molecular docking of **3e**, **3h**, **3i**, and etoposide (from left to right) towards type II topoisomerase beta (TOP2β).
